# BRAF^V600E^ drives dedifferentiation in small intestinal and colonic organoids and cooperates with mutant p53 and Apc loss in transformation

**DOI:** 10.1038/s41388-020-01414-9

**Published:** 2020-08-13

**Authors:** Nadine Reischmann, Geoffroy Andrieux, Ricarda Griffin, Thomas Reinheckel, Melanie Boerries, Tilman Brummer

**Affiliations:** 1 Institute of Molecular Medicine and Cell Research (IMMZ), University of Freiburg, Freiburg, Germany; 2 Spemann Graduate School of Biology and Medicine (SGBM), University of Freiburg, Freiburg, Germany; 3 Faculty of Biology, University of Freiburg, Freiburg, Germany; 4 Institute of Medical Bioinformatics and Systems Medicine, Medical Center—University of Freiburg, Freiburg, Germany; 5 German Cancer Consortium (DKTK), partner site Freiburg; and German Cancer Research Center (DKFZ), Heidelberg, Germany; 6 Comprehensive Cancer Centre Freiburg (CCCF), University Medical Center Freiburg, University of Freiburg, Freiburg, Germany; 7 Centre for Biological Signalling Studies BIOSS, University of Freiburg, Freiburg, Germany

**Keywords:** Colorectal cancer, Mechanisms of disease

## Abstract

BRAF^V600E^ confers poor prognosis and is associated with a distinct subtype of colorectal cancer (CRC). Little is known, however, about the genetic events driving the initiation and progression of BRAF^V600E^ mutant CRCs. Recent genetic analyses of CRCs indicate that BRAF^V600E^ often coexists with alterations in the WNT- and p53 pathways, but their cooperation remains ill-defined. Therefore, we systematically compared small and large intestinal organoids from mice harboring conditional *Braf*^*floxV600E*^, *Trp53*^*LSL-R172H*^, and/or *Apc*^*flox/flox*^ alleles. Using these isogenic models, we observe tissue-specific differences toward sudden BRAF^V600E^ expression, which can be attributed to different ERK-pathway ground states in small and large intestinal crypts. BRAF^V600E^ alone causes transient proliferation and suppresses epithelial organization, followed by organoid disintegration. Moreover, BRAF^V600E^ induces a fetal-like dedifferentiation transcriptional program in colonic organoids, which resembles human BRAF^V600E^-driven CRC. Co-expression of p53^R172H^ delays organoid disintegration, confers anchorage-independent growth, and induces invasive properties. Interestingly, p53^R172H^ cooperates with BRAF^V600E^ to modulate the abundance of transcripts linked to carcinogenesis, in particular within colonic organoids. Remarkably, WNT-pathway activation by *Apc* deletion fully protects organoids against BRAF^V600E^-induced disintegration and confers growth/niche factor independence. Still, *Apc-*deficient BRAF^V600E^-mutant organoids remain sensitive toward the MEK inhibitor trametinib, albeit p53^R172H^ confers partial resistance against this clinically relevant compound. In summary, our systematic comparison of the response of small and large intestinal organoids to oncogenic alterations suggests colonic organoids to be better suited to model the human situation. In addition, our work on BRAF-, p53-, and WNT-pathway mutations provides new insights into their cooperation and for the design of targeted therapies.

## Introduction

Colorectal cancer (CRC) represents a heterogeneous disease with distinct disease mechanisms and prognoses [1]. This heterogeneity is explained by a multistep carcinogenesis, involving the dysregulation of several signaling axes with combinations of Wnt, ERK, PI3K, TGFβ, and Notch pathway alterations. These promote tumorigenesis either through transformation of intestinal stem cells (ISC) or dedifferentiation of their progeny [2]. Frequent ERK-pathway alterations comprise *KRAS* and *BRAF* mutations, although both oncogenes trigger overlapping and distinct processes [3]. The most common BRAF mutation, the V600E substitution, generates a constitutively active oncoprotein and occurs in 11% of CRCs. BRAF^V600E^ predicts poor survival, particularly in microsatellite-stable (MSS) tumors [4].

Most BRAF^V600E^-driven CRCs arise via the so-called serrated pathway that differs from the classical adenoma–carcinoma sequence in which cancers are generated by early arising Wnt-pathway alterations followed by *KRAS*, *SMAD4*, and *TP53* mutations. This concept is corroborated by the analysis of human CRC specimen and the recapitulation of the serrated histology in mouse models [5–9].

Moreover, BRAF^V600E^-mutant CRCs predominantly occur in the proximal colon, display mucinous histology and a poor differentiation status [10–13]. Mechanistically, reduced differentiation is linked to loss of CDX2, a master transcription factor for intestinal differentiation [10, 12]. Indeed, BRAF^V600E^ signaling suppresses CDX2 expression and thereby differentiation, whereas BRAF inhibitors induce epithelial re-differentiation in human CRC cell lines [10].

BRAF^V600E^-mutant CRCs show an early and distinct metastasis pattern [13]. In contrast to the classical *APC-KRAS-TP53* sequence, BRAF^V600E^-mutant CRC is less studied. The fact that BRAF^V600E^ by itself cannot trigger metastatic disease in mice [2, 8] raises the question with which other genetic alterations it cooperates in carcinogenesis. *BRAF*-mutant CRC cell lines often carry *TP53* mutations [10], suggesting their contribution to CRC progression, as observed in other entities [14]. Indeed, several observations pinpoint to a functional relationship between *BRAF*^V600E^ and *TP53* mutations in establishing metastatic CRCs. First, Rad et al. [6] showed in their mouse model that BRAF^V600E^ is more likely to induce metastatic disease in combination with *Tp53*^R172H^, although the underlying mechanisms were not addressed. Second, 58% of serrated adenocarcinoma displayed strong nuclear p53 staining, indicating mutant p53 protein [7]. A very recent analysis showed that CRCs with co-alterations in RAS or BRAF together with TP53 mutations were associated with worse overall survival and metastasis [15]. In addition, BRAF-mutant CRCs display aberrant WNT-pathway activity. For example, increased nuclear β-catenin localization and WNT target gene expression have been observed during tumor progression in BRAF^V600E^ knock-in mice [6, 16], while WNT signaling promoting alterations, e.g, *RNF43* mutations or *RSPO3* fusions, have been recently detected in *BRAF*^*V600E*^-mutant human CRCs [17–20].

These associations raise the question how the BRAF, WNT, and p53 pathways collaborate in colorectal carcinogenesis. The many (epi)genetic alterations present in human CRCs and their cell line derivatives, however, limit the retrospective functional dissection of their individual contribution to tumor initiation and progression. Therefore, the reconstruction of these events (and their interplay) is best conducted in an oncogene naive system, such as organoids from intestinal crypts of genetically engineered mice [21, 22].

Most studies using organoids for CRC-related questions, however, induce oncogenes rather in the small intestine (SI) than in the colon (COL) [9, 11, 23–25]. The fact that SI carcinomas account for only 2% of gastrointestinal tumors [26], however, raises the question whether SI organoids represent faithful CRC models. Here, we address this question by systematically comparing the cellular behavior and transcriptomes of SI and COL organoids from knock-in mice allowing the conditional expression of BRAF^V600E^ and p53^R172H^, either singly or in combination. We show that BRAF^V600E^ expression in both organoid types leads to simultaneous processes such as the collapse of the WNT-producing ISC niche and dedifferentiation. However, restoration of WNT signaling by *Apc* deficiency rescues organoids confronted with BRAF^V600E^. Moreover, mutant p53 cooperates with BRAF^V600E^ in inducing prerequisites for metastasis and in recapitulating human CRC signatures.

## Results

### BRAF^V600E^ affects the organization of SI and COL organoids

To study the consequences of BRAF^V600E^ and/or p53^R172H^ expression in an oncogene naive setting, we crossed *Braf*^floxV600E^ or *Trp53*^LSL-R172H^ knock-in mice with *Villin::CreER*^*T2*^ transgenic animals expressing the 4-hydroxy-tamoxifen (4-HT)-regulated Cre recombinase under the control of the *Villin* promoter [27] (Supplementary Fig. S1a). In the absence of Cre activity, the *Braf*^floxV600E^ allele ensures expression of wild-type BRAF [28], while *Trp53*^LSL-R172H^ contains a *lox*P-STOP-*lox*P cassette preventing p53^R172H^ expression [29]. The R172H substitution confers dominant-negative effects and corresponds to the R175H mutation found in human tumors, incl. CRCs [14]. Supplementary Fig. S1b demonstrates the efficient 4-HT induced Cre-mediated recombination of the *Braf* and *Trp53* loci in both SI and COL organoids. BRAF^V600E^ expression and its downstream effects on ERK-pathway activation were confirmed by western blotting (Supplementary Fig. S1c).

Previous studies using either SI or COL organoids noted that BRAF^V600E^ induces organoid disintegration and cell death. However, this was not observed by others (summarized in Supplementary Table S1), albeit different experimental approaches were applied, and no study has yet compared organoids from both tissues side-by-side. Here, we show that BRAF^V600E^ caused an initial rapid size expansion in both organoid types, followed by their disintegration and cell death (Supplementary Fig. S1d–g and Supplementary Videos S1, 2). Interestingly, our comparison uncovered that COL organoids disintegrated earlier than their SI counterparts, and that disintegration was slightly but significantly delayed in COL organoids by co-expression of p53^R172H^ (Supplementary Fig. S1e). Neither induction of p53^R172H^ alone (Supplementary Fig. S2a) nor 4-HT treatment of organoids lacking floxed alleles or CreER^T2^ triggered disintegration (Supplementary Fig. S2b), confirming that this phenotype is specifically caused by BRAF^V600E^.

The disintegration of BRAF^V600E^ expressing organoids prompted us to analyze their epithelial organization in more detail. As described in Supplementary Fig. S2c–f, BRAF^V600E^ induced proliferation outside of morphologically defined ISC niches, irregularities in epithelial organization, impaired tight junction function, and loss of the stem cell niches. In summary, our BRAF^V600E^ knock-in approach confirms previous findings on SI organoids showing a profound impact of transgenic BRAF^V600K^ or BRAF^V600E^ on organoid organization [9, 30] and demonstrates for the first time that BRAF^V600E^ affects similar processes in COL organoids.

### BRAF^V600E^ and p53^R172H^ collaborate in inducing transcriptomic changes reminiscent of human CRC

**Fig. 1:**
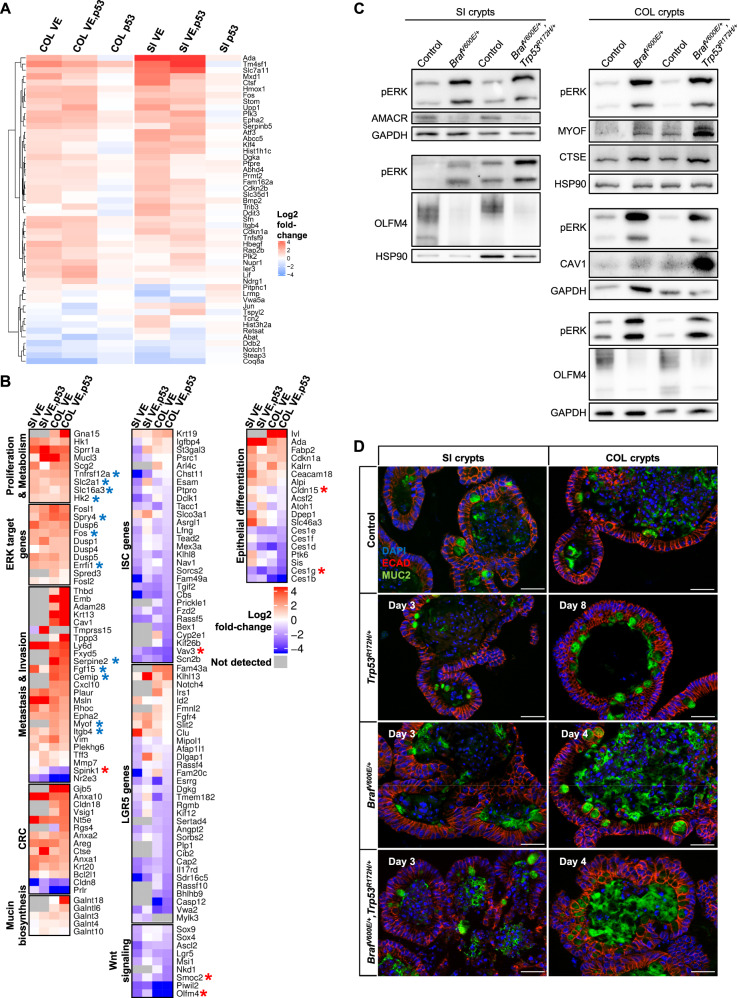
RNA-Seq of *Braf*^*V600E/+*^ (VE) and *Trp53*^*R172H/+*^ (p53) single and *Braf*^*V600E/+*^*,Trp53*^*R172H/+*^ (VE,p53) double-mutant SI and COL organoids (GSE132551). **a** Heatmap of the log2 fold changes (induced vs. non-induced, color coded) of the genes listed in the HALLMARK_P53_PATHWAY MSigDB gene set. **b** Heatmap of selected differentially regulated transcripts. Color code represents the log2 fold change (induced vs. non-induced). Asterisks marked genes have already been found to be among the top 50 of differentially regulated genes in human CRC cell line spheroids upon BRAF^V600E^ inhibition ([10]; blue = downregulated; red = upregulated). **c** Western blots (WB) of SI and COL organoids using the indicated antibodies. GAPDH and HSP90 serve as loading controls. Each subpanel identified by its pERK detection reflects a distinct biological experiment. **d** MUC2 IF staining of formalin-fixed paraffin-embedded (FFPE) SI and COL organoid sections shows enhanced mucin production within mutant organoids, with highest levels in double-mutant ones. Scale bars: 50 µm.

Following the analysis of BRAF^V600E^ (and p53^R172H^)-induced changes in SI and COL organoids, we assessed the impact and functional relationship of the two oncogenic mutations in both organoid types by RNA sequencing (RNA-Seq). The principle component analysis (PCA) shows that the gene expression profiles of SI and COL organoids significantly differ in their oncogene naive ground state (Supplementary Fig. S3a). As expected from their phenotypes, organoids expressing BRAF^V600E^, either singly or in combination with p53^R172H^, exhibited strong changes in their transcriptomes, while mutant p53 alone had only little impact on gene expression. Gene set enrichment analysis (GSEA) confirmed that p53^R172H^-mutant organoids displayed altered expression of p53 pathway genes, indicating successful loss of p53 wild-type function (Fig. 1a). Oncogene induction in BRAF^V600E^ and BRAF^V600E^/p53^R172H^ organoids induced a strong p53 signature; however, diminished p53 target gene activation could still be detected within the double-mutant organoids. Figure 1b compares the relative fold changes of selected transcripts upon induction of BRAF^V600E^, alone or in combination with p53^R172H^, in SI and COL organoids. In both organoid types, BRAF^V600E^ induced transcripts associated with metabolic rewiring (e.g., the glycolytic key enzymes HK1/2) as well as an immediate early gene response as reflected by the FOS family transcription factors (*Fosl1*, *Fos*) and negative feedback regulators of the EGFR/RAS/RAF/MEK/ERK pathway, such as *Dusp4/5/6*, *Spry4*, and *Errfi1*. Of note, these and other transcripts were shown to be reversely affected by BRAF inhibitors in human CRC cell lines [10, 31], and their cross-validation in clinical CRC samples and prognostic relevance is presented in a literature survey in Supplementary Table S2. Many transcripts positively associated with invasion and metastasis were upregulated in both organoid types. Interestingly, several of them displayed a more pronounced fold change in COL than in SI organoids, or were exclusively altered in COL organoids. Examples for this category are *Myof* [32] and *Fgf15*, whose human orthologue FGF19 has been linked to CRC aggressiveness [33] (Fig. 1b, c). A switch to pro-metastatic processes becomes further evident by the upregulation of β-integrin subunits such as *Itgb4* (Fig. 1b) and ITGB1, which is recruited to the organoid plasma membranes (Supplementary Fig. S3b) [34].

Likewise, BRAF^V600E^-mutant organoids more abundantly expressed transcripts associated with human CRC and, in line with the mucinous phenotype of human BRAF^V600E^-positive tumors, mucin biosynthesis (Fig. 1b). Indeed, BRAF^V600E^-expressing organoids secreted Mucin2 (Muc2) into their lumina, while expression of this glycoprotein was largely confined to Goblet cells in control and p53^R127H^-positive organoids (Fig. 1d). The upregulation of Cathepsin E (*Ctse*), a protease that is strongly associated with serrated adenoma [35, 36], represents another parallel to human CRCs (Fig. 1b, c; Supplementary Fig. S3c). Moreover, genes like *Cldn18* and the two cell-junction protein genes *Gjb5* and *Vsig1*, which all have been associated with the serrated pathway [36, 37], were only detected in COL organoids and especially upregulated in the double-mutant ones (Fig. 1b). This is in line with a highly significant correlation of the BRAF^V600E^-expressing COL organoids with gene expression profiles from human sessile-serrated adenoma (SSA) patients (Supplementary Fig. S4).

Given that BRAF^V600E^ has been implicated in ISC exhaustion in SI organoids [9, 11], we were interested whether BRAF^V600E^ (and p53^R172H^) would affect transcripts associated with intestinal stemness and the Lgr5/Wnt-signaling pathway. As shown in Fig. 1b, BRAF^V600E^ suppressed many transcripts, which have been linked to ISC biology [38], in both organoid types. This includes, the ISC niche markers *Olfm4* and *Smoc2*, which have been linked to Wnt signaling [39] (Fig. 1b, c), and supports the aforementioned morphological observation that the integrity of the ISC niche is lost (Supplementary Figs. S1d and S2f).

Moreover, induction of BRAF^V600E^ also decreased intestinal differentiation markers, in particular in COL organoids (Fig. 1b). This agrees with our previous study showing that BRAF^V600E^ inhibitors induce epithelial differentiation markers such as Claudin-15, AMACR, and carboxyesterases (Ces) in human CRC cell lines [10]. The latter two as well as *Sis*, encoding a sucrase isomaltase downregulated by BRAF^V600E^ in COL organoids, are well-known CDX2 target genes [40, 41]. This suggests that BRAF^V600E^ also counteracts epithelial differentiation in murine oncogene naive organoids.

In summary, RNA-Seq identified profound differences between SI and COL organoids upon oncogene expression. Importantly, our side-by-side comparison revealed that the BRAF^V600E^-mutant COL organoids more abundantly express transcripts associated with human CRC.

### Oncogene naive SI and COL crypts significantly differ in ERK-pathway activity

**Fig. 2:**
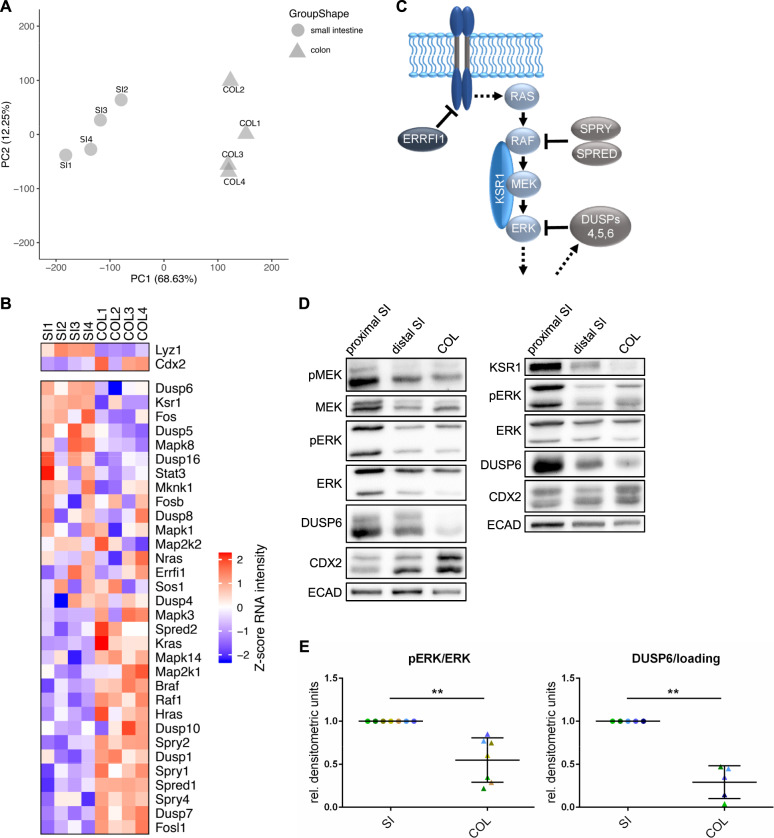
Primary small and large intestine exhibit differences in basal ERK-signaling activity (GSE132546). **a** PCA of RNA-Seq of freshly isolated small intestinal (SI, circles) and colonic (COL, triangles) crypts from two female (no. 1, 4) and two male (no. 2, 3) donor mice. SI and COL show a clear separation according to PC1. **b** Heatmap of transcripts encoding ERK-pathway components shows differential expression between SI and COL crypts freshly isolated from the four donor mice described in **a**. Color code represents the row-wise scaled (*Z* score) RNA intensity. **c** Simplified cartoon visualizing ERK-pathway components of particular interest. **d** WB of SI and COL crypts with the indicated antibodies. E-cadherin serves as loading a control. Of note, RNA-Seq revealed that other loading controls, such as GAPDH or beta-actin were differentially expressed between SI and COL crypts. Only E-cadherin displayed a negligible log2 fold change of 0.004. **e** Quantification of WB analyses of pERK and DUSP6. ERK phosphorylation was normalized to total ERK expression, and DUSP6 was normalized to internal loading control. Green/brown colors indicate whole-tissue lysates, while blue colors indicate isolated crypt lysates. Each color depicts one donor mouse. Data are presented as mean ± SD. **P ≤ 0.01 (paired *t* test, *n* ≥ 3 donor mice).

The marked differences between SI and COL organoids in response to oncoprotein expression prompted us to compare the transcriptomes of primary intestinal crypts. As shown by the PCA in Fig. 2a, SI and COL crypts significantly differed in their transcriptomic ground state. Importantly, crypts from both tissues displayed unanticipated differences in the expression of ERK-pathway elements and target genes (Fig. 2b–e). For example, KSR1, a potent RAF activator and scaffolding protein for the three core kinases of the RAF/MEK/ERK module [42, 43], was more highly expressed at the RNA and protein level in SI organoids. Commensurate with previous studies in other cell types (see references [42, 43] and references therein), we demonstrate a positive correlation between KSR1 expression and MEK/ERK phosphorylation for intestinal tissue (Fig. 2d, e). Of note, KSR1 expression, MEK/ERK phosphorylation, and expression of the ERK target gene DUSP6 were highest in proximal SI, the tissue usually used for organoid generation [21] (Fig. 2d). In contrast, but as expected from previous observations [44], Cdx2 expression increased along the anterior–posterior axis. Interestingly, Spred and Sprouty (Spry), which suppress the ERK pathway at its apex [45], were also more abundantly expressed in COL crypts. These observations indicate that the ERK axis is more stringently controlled in the COL, and that higher pathway activity is tolerated in the SI.

### BRAF^V600E^ induces a fetal signature in murine organoids that closely resembles transcriptome profiles of human BRAF-mutant CRCs

**Fig. 3:**
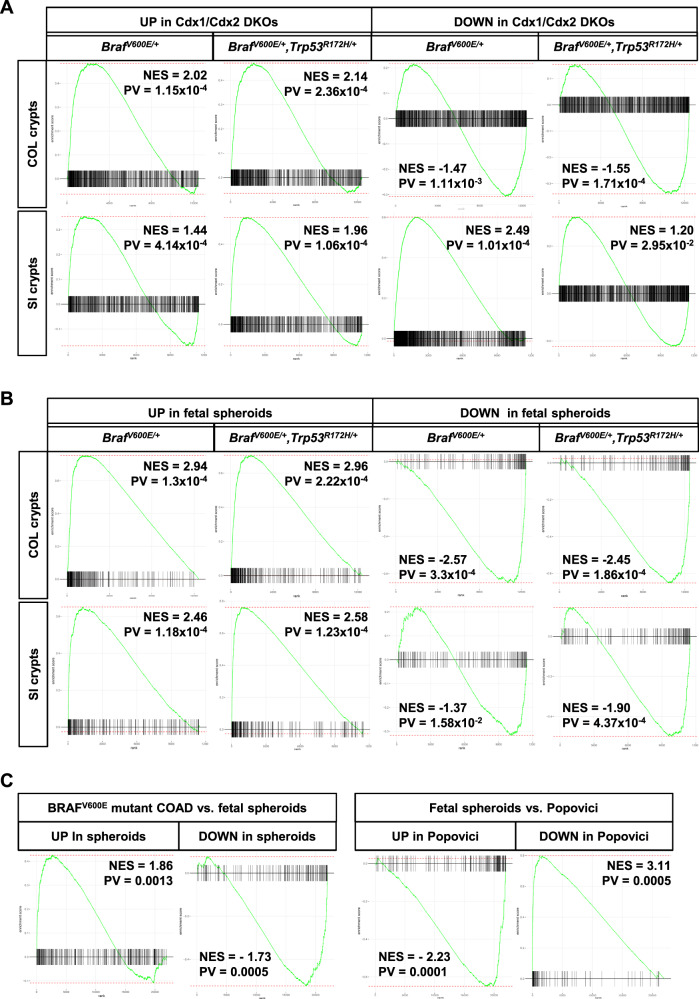
BRAF^V600E^ induces a fetal signature in COL organoids. **a** Gene set enrichment analysis (GSEA) of our transcriptomic data with genes that are up- (left) and downregulated (right), respectively, in *Cdx1/Cdx2* double-knockout (DKO) mice (GSE24633). **b** GSEA of our transcriptomic data against genes that are up- (left) and downregulated (right), respectively, in mouse fetal intestinal spheroids. **c** GSEAs showing the comparison of the LGR5-independent fetal signature to human data sets. The left panel shows the enrichment of the fetal spheroids signature in TCGA BRAF^V600E^ mutant vs. healthy colon. The right panel shows the enrichment of the Popovici signature, i.e., 314 differentially expressed probe sets between WT and BRAFm samples, in fetal spheroids. Note, a positive fold change of the “Popovici genes” indicates higher expression in WT vs BRAFm, which results in an inverse correlation to the fetal signature. NES normalized enrichment score, PV *P*-value.

Interestingly, our RNA-Seq analysis revealed that BRAF^V600E^ triggers processes linked to both ends of the functional crypt–villus axis. On the one hand, we confirm ISC niche exhaustion by the loss of Paneth cells in SI organoids (Supplementary Fig. S2f) and the decrease of transcripts associated with intestinal stemness and Lgr5/Wnt signaling (Fig. 1b, c). On the other hand, induction of BRAF^V600E^ also markedly reduced intestinal differentiation markers, in particular in COL organoids (Fig. 1b, c). This is further supported by Fig. 3a, demonstrating a strong enrichment of the intestine data sets from *Cdx1/Cdx2* double-knockout mice (GSE24633) [46] in our COL organoid transcriptomes. Of note, especially the BRAF^V600E^/p53^R172H^ double-mutant COL organoids resembled the *Cdx1/Cdx2* knockout signature. This agrees with the stronger reduction of CDX2 target genes, like *Sis* and *Ces* [40, 41], as well as with stronger upregulation of *Cldn18*, which has been negatively correlated with CDX2 expression and associated with poor survival in CRC patients [47] (Fig. 1b).

In 2013, Mustata et al. identified Lgr5-independent progenitors of murine fetal intestines, which give rise to poorly differentiated spheroids displaying phenotypical characteristics that profoundly differ from those of adult ISCs [48]. Similar to the phenotype observed in BRAF^V600E^-expressing organoids (Supplementary Fig. S2c, f), the fetal spheroids presented with no Paneth cells and proliferating cells, which were not restricted to the crypt bases. Importantly, their transcriptome significantly differed from adult organoids with only a very small overlap in WNT target gene expression and a poor differentiation status. This prompted us to compare our transcriptomic data with the genes that are up- and downregulated in fetal spheroids, respectively (Fig. 3b). Indeed, the GSEA demonstrates a clear positive correlation of our organoid and the fetal spheroid transcriptomes, since up- and downregulated genes in fetal spheroids are enriched and depleted, respectively. This indicates that BRAF^V600E^ and BRAF^V600E^/p53^R172H^ induce a fetal spheroid-like program within intestinal organoids isolated from adult mice. Interestingly, there is a higher similarity between the transcriptomes of COL organoids to fetal spheroids, although Mustata et al. [48] used embryonic SI as an experimental model. Next, we asked whether the fetal signature is also relevant for human CRC. First, we compared 54 BRAF^V600E^-mutant colorectal adenocarcinoma (COAD) to 41 healthy colon samples listed in the TCGA database and then calculated the correlation to the LGR5-independent fetal signature (Fig. 3c, left GSEAs). Indeed, we observe a highly significant correlation between the fetal signature and BRAF^V600E^-mutant COAD. Second, we demonstrate a highly significant inverse correlation between the fetal signature and the 314 differentially expressed probe sets distinguishing BRAF wild-type and mutant samples identified by Popovici et al. (Fig. 3c, right GSEAs (Popovici et al. [49])). Thus, two independent approaches support the operation of a BRAF^V600E^-induced fetal dedifferentiation program in human CRC, which is recapitulated by our murine organoids.

### p53^R172H^ confers invasive properties to BRAF^V600E^ expressing COL organoids

Our above-described RNA-Seq analysis of BRAF^V600E^ and/or p53^R172H^-mutant COL organoids revealed that 72 out of the 129 selected BRAF^V600E^-responsive transcripts, outside of the ERK target gene category, were more strongly modulated when p53^R172H^ was present (Fig. 1b). Interestingly, several of them are linked to tumor progression. Validation of the RNA-Seq analysis revealed that co-expression of p53^R172H^ further suppressed protein expression of NR2E3, which emerged as a potential tumor suppressor in breast and liver cancer [50, 51] (Supplementary Fig. S3d). Conversely, Ephrin type-A receptor 2 (EPHA2), a transcriptional RAS/RAF target [52], which is overexpressed in several human cancers, including CRC [53, 54], was more strongly upregulated at the protein level when p53^R172H^ was present (Supplementary Fig. S3d). Similarly, co-expression of p53^R172H^ increased expression of CTSE (Fig. 1c; Supplementary Fig. S3c, e) and Caveolin-1 (CAV1) (Fig. 1c; Supplementary Fig. S3e). Although its role in metastasis formation is controversially discussed [55], there is evidence that CAV1 is decreased in early stages of carcinogenesis [56, 57], but is found to be elevated at later stages [58], including in CRC [59]. Moreover, increased CAV1 expression in T4-stage CRCs was associated with increased invasiveness [60], and depletion of CAV1 reduced the invasive properties of human CRC cell lines [61].

**Fig. 4:**
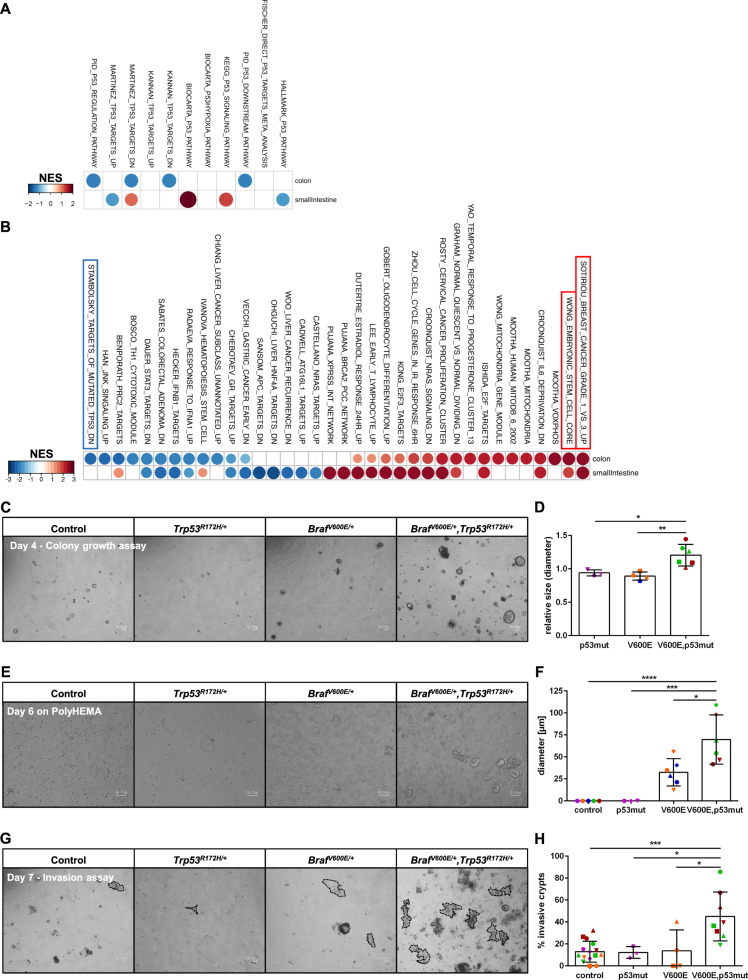
*Trp53*^*R172H*^ supports proliferative and invasive characteristics of *Braf*^*V600E*^ mutant organoids. **a**, **b** GSEA of the delta log2 fold changes of double-mutant vs. BRAF^V600E^-only organoids was performed. Shown are enrichment heatmaps for p53-related gene sets (**a**) and for the top ten significantly (*P* < 0.05) regulated chemical and genetic perturbations (CGP) (**b**). On both heatmaps, color code and circle size represent NES. **c** Representative bright-field (BF) images of three independent experiments show colony growth capacity of COL organoids. Quantification is shown in (**d**), **d** Quantification of colony growth capacity, normalized to the corresponding non-induced control. The longest straight lines of the crypts were measured. **e** Control or 4-HT-induced COL crypts were disaggregated, and grown on PolyHEMA-coated culture dishes for 6 days before BF images were taken and the diameters of the formed cell clusters were measured. Representative BF pictures of ≥3 independent experiments that are quantified in (**f**), are shown. **f** Quantification of anchorage-independent growth. The longest straight lines of the cell clusters were measured. Note that neither non-induced controls nor p53^R172H^-mutant organoids were able to form cell clusters on PolyHEMA. **g** Control or 4-HT-induced COL organoids were grown in diluted (50%) Matrigel. BF images were taken at day 7, and organoids attached to the plastic surface were counted (highlighted by dashed lines). Representative pictures of ≥3 independent experiments are shown, which are quantified in (**h**). Higher-magnification BF images of “invaded” organoids are shown in Supplementary Fig. S7. **h** Quantification of “invaded” organoids. In (**c**, **e**, **g**), scale bars: 50 µm. In (**d**, **f**, **h**), symbol colors refer to donor mice, symbol shapes refer to independent experiments. Data are presented as mean ± SD, and statistical significance was determined by one-way ANOVA (corrected for multiple comparison by Bonferroni). *P ≤ 0.05; **P ≤ 0.01; ***P ≤ 0.001; ****P ≤ 0.0001.

All these observations already support the collaboration between BRAF^V600E^ and p53^R172H^. To further investigate the functional relationship of the two oncogenic mutations, we introduced the delta log2 fold change between the double-mutant and BRAF^V600E^-only organoids and subjected it to GSEA. Of note, we observed decreased activation of p53-related gene signatures (Fig. 4a) and the downregulation of mutated p53 target genes (STAMBOLSKY_TARGETS_OF_MUTATED_TP53) only within the COL organoids (Fig. 4b). This further highlights tissue-specific reactions toward oncogenic stress. Furthermore, the top 50 significantly regulated Molecular Signatures Database (MSigDB) gene sets revealed the activation of several mitochondria, cellular respiration, and cell cycle signatures, indicating enhanced proliferation and metabolic activity upon co-expression of p53^R172H^ (Supplementary Fig. S5). Interestingly, the double-mutant organoids show an increased expression of genes associated with invasive grade 3 breast cancer [62] (SOTIRIOU_BREAST_CANCER_GRADE_1_VS_3_UP), as well as of genes that are part of the embryonic stem cell signature (WONG_EMBRYONIC_STEM_CELL_CORE), which is activated in epithelial tumors that are likely to progress to metastasis [63] (Fig. 4b). Furthermore, COL organoids also exhibited decreased expression of genes, which are downregulated in invasive mammary ductal carcinoma (SCHUETZ_BREAST_CANCER_DUCTAL_INVASIVE_DN) (Supplementary Fig. S5b). The activation of these invasion-associated signatures by the addition of p53^R172H^, especially within the COL organoids, ties in with the higher expression of selected “metastasis & invasion” associated genes (Fig. 1b). It should be noted, however, that, although some genes from the EMT hallmark classifier were more strongly induced in organoids co-expressing BRAF^V600E^ and p53^R172H^ (Supplementary Fig. S6), we did not observe a “classical” epithelial-to-mesenchymal transition (EMT), as defined by the loss of E-cadherin or the induction of transcription factors like ZEB1/2, SNAIL, SLUG, or TWIST in organoids.

As RNA-Seq further revealed that BRAF^V600E^/p53^R172H^-expressing COL organoids display a transcriptional signature reflecting processes contributing to tumor progression and metastasis, we set up three experimental assays that reflect key prerequisites for tumor cell dissemination. First, we addressed the colony growth capacity after disruption into single cells (Fig. 4c, d). Interestingly, BRAF^V600E^/p53^R172H^ double-mutants formed larger colonies than those expressing either BRAF^V600E^ or p53^R172H^. Anchorage-independent growth represents a stringent key characteristic of transformed epithelial cells and prerequisite for metastasis. Therefore, cells derived from COL organoids were plated on culture dishes coated with poly(2-hydroxyethyl methacrylate) (PolyHEMA), a polymer preventing vessel adhesion. As expected, cells from control and p53^R172H^-mutant organoids did not thrive under these conditions, and cells expressing BRAF^V600E^ showed only little growth. The BRAF^V600E^/p53^R172H^ double-mutants, however, displayed increased anchorage-independent growth (Fig. 4e, f) and, furthermore, presented with invasive behavior in a diluted Matrigel matrix (Fig. 4g, h; Supplementary Fig. S7) [25]. These observations are in line with the reported function of p53 as inducer of anoikis, a programmed cell death upon anchorage-independent conditions, which needs to be overcome by cancer cells during cancer progression and metastatic colonization [64, 65]. Indeed, BRAF^V600E^/p53^R172H^ double-mutant organoids displayed higher CAV1 expression than the BRAF^V600E^ single-mutants (Fig. 1b, c; Supplementary Fig. S3e), which is in line with its reported role in mediating anoikis resistance in several cancer entities [66–68]. Collectively, these data support the metastasis-associated transcriptomic signatures and further imply that p53^R172H^ promotes the fitness and invasive properties of BRAF^V600E^-expressing organoids, which supports the assumption that *TP53* mutations are important for the transition from adenoma to carcinoma [69, 70].

### *Apc* inactivation allows BRAF^V600E^ expressing cells to establish long-term surviving organoids

**Fig. 5:**
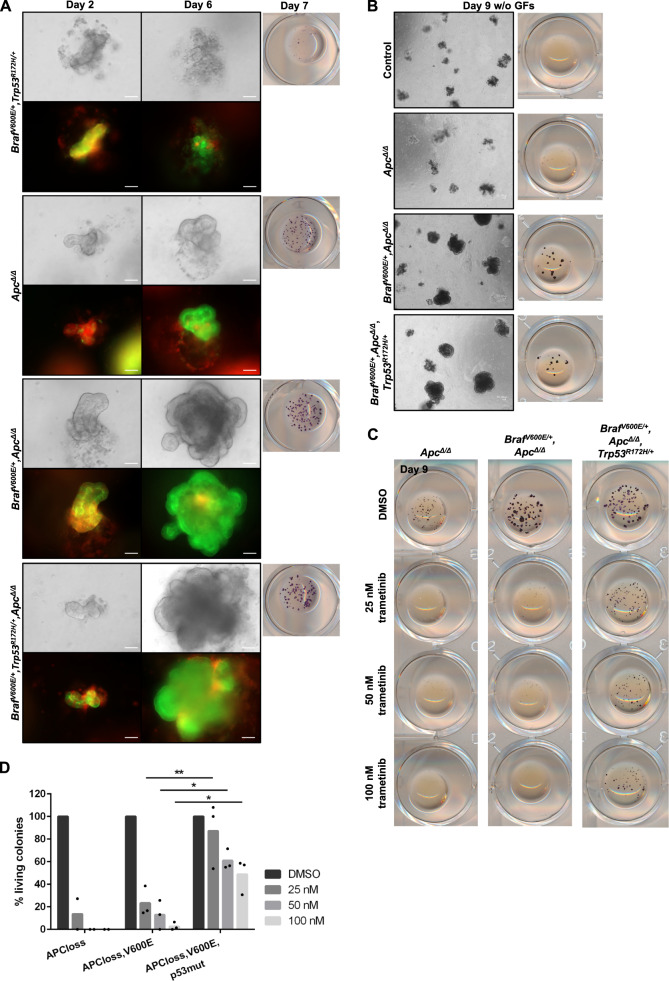
Loss of APC rescues the BRAF^V600E^-driven disintegrative phenotype and confers growth factor independence. **a** COL organoids with the indicted genotypes were treated with 3 µM 4-HT for 24 h. Representative microscopy pictures at days 2 and 6 (see also Supplementary Video 3) and MTT staining at day 7 are shown. **b** COL organoids with the indicated genotypes were induced with 3 µM 4-HT and cultured without the growth factors (GFs) EGF, R-Spondin, Noggin, and Wnt3a. BF images and MTT staining at day 9 are shown. **c** COL organoids with the indicated genotypes were treated with DMSO or indicated trametinib concentrations 1 day after induction with 3 µM 4-HT. Representative MTT staining at day 9 after 4-HT induction is shown. Note that the dark-blue colonies indicate metabolic activity. **d** Quantification of trametinib treatment shown in (**c**) of two (for *Apc*^*Δ/Δ*^) and three (for *Braf*^*V600E/+*^*,Apc*^*Δ/Δ*^ and *Braf*^*V600E/+*^*,Apc*^*Δ/Δ*^*,Trp53*^*R172H/+*^*)* independent experiments. Colony count was normalized to corresponding DMSO control, and statistical significance was determined by two-way ANOVA (corrected for multiple comparison by Bonferroni). *P ≤ 0.05; **P ≤ 0.01. In (**a**, **b**), scale bars: 50 µm.

**Fig. 6:**
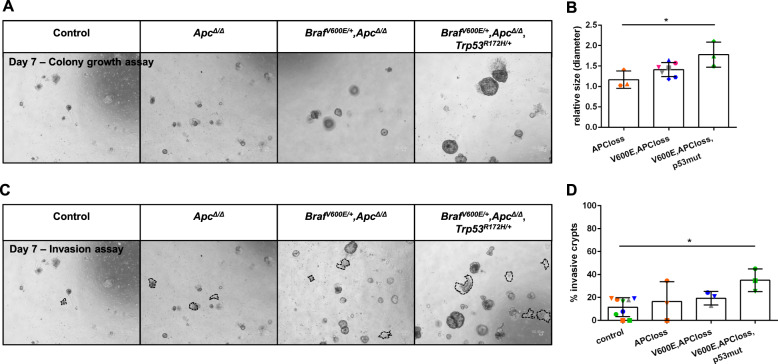
*Trp53*^*R172H*^ supports proliferative and invasive characteristics of *Braf*^*V600E/+*^*,Apc*^*Δ/Δ*^ mutant organoids. **a** Representative BF images of COL organoid colony growth capacity of ≥3 independent experiments are shown, which are quantified in (**b**). **b** Quantification of colony growth assay, normalized to corresponding non-induced control. The longest straight lines of the crypts were measured. **c** COL organoids with the indicated genotypes were grown in diluted (50%) Matrigel. BF images were taken at day 7, and organoids attached to the plastic surface were counted (highlighted by dashed lines). Representative pictures of three independent experiments are shown, and are quantified in (**d**). **d** Quantification of “invaded” organoids. In (**a**, **c**), scale bars: 50 µm. In (**b**, **d**), symbol colors refer to donor mice, symbol shapes refer to independent experiments. Data are presented as mean ± SD, and statistical significance was determined by one-way ANOVA (corrected for multiple comparison by Bonferroni). *P ≤ 0.05.

Given that Wnt target genes are strongly reduced in BRAF^V600E^ expressing COL organoids, and that survival of SI organoids is augmented by pharmacological or ligand-induced WNT-pathway activation [9, 11], we tested whether this phenomenon is also applicable to COL organoids. Indeed, the GSK3 inhibitor CHIR-99021 improved the survival of BRAF^V600E^ expressing COL organoids (Supplementary Fig. S8a). In addition to promoting β-catenin degradation and thereby suppressing canonical Wnt signaling, GSK3 is involved in many other cellular processes [71], and hence this pharmacological approach by itself does not provide enough mechanistic support for a protection against BRAF^V600E^-induced organoid disintegration by Wnt signaling. Therefore, we asked whether genetic WNT-pathway activation would increase the fitness of BRAF^V600E^ expressing COL organoids. To this end, we crossed *Braf*^*floxV600E*^; *Villin::CreER*^*T2*^ mice (with or without the *Trp53*^LSL—R172H^ allele) with animals harboring the conditional *Apc*-knockout allele [72] and the *Rosa26::mTOM/mGFP* Cre reporter [73]. Cre-mediated recombination occurred very efficiently as reflected by the recombination of the *Apc* locus and the switch to mGFP expression (Supplementary Fig. S8b, c). APC loss successfully induced WNT-pathway activation, as indicated by OLFM4 preservation within *Braf*^*V600E/+*^*,Apc*^*Δ/Δ*^ COL organoids (Supplementary Fig. S8d). Interestingly, genetic Wnt-pathway activation overcame the need for an intact ISC niche as the organoids continued to proliferate without any disintegration (Fig. 5a; Supplementary Video S3), although APC deficiency neither affected ERK phosphorylation (Supplementary Fig. S8d) nor restored tight junction function (Supplementary Fig. S8e). Next, we aimed at defining the growth/niche factor requirements of our COL organoid series (Fig. 5b). While APC loss alone could not prevent organoid death in the absence of the growth factors (GF) EGF, R-Spondin, Noggin, and Wnt3a, its combination with BRAF^V600E^ or BRAF^V600E^/p53^R172H^ conferred strong GF independence. This prompted us to define whether BRAF^V600E^-derived signals were still required in the context of APC inactivation. Therefore, we incubated these APC-deficient BRAF^V600E^ expressing organoids with increasing doses of trametinib, a highly selective MEK inhibitor clinically applied in *BRAF*-mutant CRC [74]. Interestingly, trametinib impaired growth of the double- and triple-mutant COL organoids, while APC-deficient ones already died at the lowest trametinib concentration, indicating that the BRAF/MEK axis still acts as the driver of proliferation (Fig. 5c, d). Surprisingly, the triple-mutant COL organoids continued to grow at the highest concentration (Fig. 5c, d), indicating increased resistance toward trametinib treatment. Therefore, we investigated whether ERK-pathway activity remains upregulated in these organoids by assessing the phosphorylation status of ERK1/2, the ERK-mediated phosphorylation of the immediate early gene products FOS and FRA1 (FOSL1), as well as total FOS expression and the expression level of the ERK target gene product DUSP6 [75]. The phosphorylation status of FOS and FRA1 serves as an excellent readout for long-term persistence effects of ERK-pathway activity [76, 77]. As shown in Supplementary Fig. S9, however, none of these read-outs were significantly elevated by p53^R172H^ in trametinib-treated organoids, indicating that mutant p53 cannot counteract acute drug-induced ERK-pathway inhibition. Nevertheless, we observed a strong trend for higher phosphorylation of ERK and FOS and, although to a lesser extent, increased FOS and DUSP6 expression in the triple-mutant organoids under steady-state conditions (Supplementary Fig. S9). This raises the possibility that p53^R172H^ could induce ERK-mediated processes that pre-adapt triple-mutant organoids to trametinib treatment und other stressors. Indeed, the triple-mutant organoids displayed increased fitness as reflected by enhanced colony-forming capacity after single-cell disruption (Fig. 6a, b) and invasive growth in diluted matrigel (Fig. 6c, d). In summary, the improved stress resistance of the triple-mutant organoids is probably best explained by the pleiotropic effects of mutant p53 on stress and cell death pathways [78]. The identification of the precise mechanisms by which p53^R172H^ confers trametinib resistance represents an area for future studies.

In summary, the profound effect of APC deficiency on organoid survival highlights the importance of an intact stem cell niche for nascent tumor cells and support our hypothesis that BRAF^V600E^ and p53^R172H^ cooperate in colorectal carcinogenesis by conferring survival signals, MEK inhibitor resistance, and invasive properties.

## Discussion

In this study, we have systematically compared the effects of BRAF^V600E^ and p53^R172H^, either singly or in combination, on oncogene naive organoids from the small and large intestine of knock-in mice. Our conditional, isogenic approach demonstrates that BRAF^V600E^ expressing SI and COL organoids undergo an initial burst of proliferation and rapidly disintegrate with the majority of cells dying. Previous work on SI organoids revealed that pharmacological inhibition of the BRAF–MEK–ERK axis prevents disintegration, indicating that this process is driven by aberrant ERK activity [9, 30]. Of note, we demonstrate that COL organoids disintegrate faster than SI-derived ones, suggesting important differences between both organoid types. Indeed, our RNA-Seq analysis shows that the transcriptomes of SI and COL primary crypts strongly differ in their oncogene naive ground state. In particular, we uncovered that ERK-pathway activity is significantly higher in SI than in COL crypts. This suggests that the latter are less adapted to high ERK levels and consequently less equipped to a rise in pathway activity. Importantly, significant differences in the transcriptional profiles between SI and COL organoids were also observed upon oncogene induction, which emphasizes the tissue-specific reaction toward sudden oncogene expression and represents important information for the design of experiments.

**Fig. 7:**
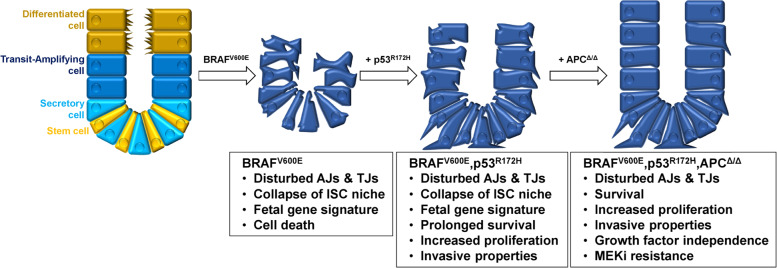
Graphical illustration summarizing the model of the multistep colorectal carcinogenesis investigated in this study. From left to right: Expression of oncogenic BRAF^V600E^ induces a fetal gene signature in adult wildtype colonic organoids, but also leads to rapid disintegration and subsequent cell death. Co-expression of p53^R172H^ extends organoid survival and conveys proliferative and invasive properties. Additional loss of APC prevents the collapse of the intestinal stem cell (ISC) niche, thereby promoting the survival of the mutant organoids. Importantly, additional APC loss confers growth factor independence and modulates the sensitivity to MEK inhibitor (MEKi) treatment. AJ adherens junctions, TJ tight junctions.

As shown in Supplementary Table S1, several groups reported with similar but not identical approaches contradictory effects of BRAF^V600E^ on the viability and integrity of intestinal organoids. We and others [9, 11, 30, 79] demonstrate that BRAF^V600E^ impairs the integrity of intestinal organoids by the exhaustion of ISCs, which provide critical niche factors (Fig. 7). This is thought to induce sudden “default” differentiation of immature cells and might reflect a defense mechanism against malignant transformation [9, 11]. At the same time, however, we observe the induction of a gene expression pattern similar to that of *Cdx1/Cdx2* double-knockout mice [46], indicating dedifferentiation. This is in line with our previous study on human CRC cell lines showing that BRAF^V600E^ depletion or inhibition leads to differentiation, in part through increasing CDX2 levels [10]. This ties in with recent studies identifying *Cdx2* as an important suppressor of BRAF^V600E^-driven CRC transformation [7, 11].

Our transcriptomic analyses indicate that both at first sight contrasting mechanisms, namely the loss of Lgr5/Wnt target gene expression in combination with the induction of dedifferentiation patterns, operate simultaneously upon BRAF^V600E^ expression. Surprisingly, this resembles the transcriptional profile of fetal intestine spheroids [48], which we found to be recapitulated in human BRAF^V600E^-mutant CRC samples. Recently deposited work implicated the YAP/TAZ pathway in the induction of the fetal signature of organoids expressing an RSPO3 fusion oncoprotein along with KRAS^G12D^ and loss of p53 [17]. Interestingly, we also observed a marked upregulation of several members of the Hippo target gene signature [80] in COL organoids, even by BRAF^V600E^ alone (Supplementary Fig. S10). In summary, we suggest that sudden BRAF^V600E^ expression in organoids causes an imbalance of the stem-to-differentiation homeostasis and a poorly differentiated phenotype.

The genetic complexity of BRAF^V600E^-mutant CRC not only aggravates their treatment but also their reconstruction in carcinogenesis models. As outlined above, BRAF^V600E^ itself is insufficient to trigger transformation and needs to cooperate with other (epi)genetic alterations. Inspired by the genotypes of commonly used BRAF^V600E^-mutant CRC cell lines [10], which carry p53 and/or WNT-pathway mutations and represent prime examples for complete niche autonomy, we asked whether these alterations would rescue BRAF^V600E^-induced organoid disintegration. Here, we report the first analysis of the cooperation between BRAF^V600E^ and p53^R172H^ in SI as well as COL organoid models. As mutant p53 contributes to escape from cell death, senescence, or genomic stress [14, 81], we speculated that p53^R172H^ helps to overcome the oncogenic stress of organoids with BRAF^V600E^ expression. Indeed, p53^R172H^ slightly extended survival of BRAF^V600E^ expressing organoids and conveyed several key properties of metastatic cells, such as increased fitness, invasive behavior and anoikis resistance (Fig. 7). This might explain the association and collaboration between *BRAF* and *TP53* mutations in metastatic CRC. Despite these advantages, however, p53^R172H^ could not prevent organoid disintegration.

In contrast, Wnt-pathway activation by bi-allelic *Apc* deficiency alone prevented the disintegrative phenotype and conferred growth/niche factor independence to BRAF^V600E^ expressing COL organoids (Fig. 7). Of note, most human BRAF^V600E^-mutant CRCs frequently display aberrant WNT-pathway activity due to the expression of R-Spondin fusion proteins or the loss of its negative regulators [17–20, 82, 83]. These alterations represent alternative mechanisms to *APC* truncations, which were originally thought to be less common in BRAF^V600E^ mutant CRC [3]. While our paper was under revision, however, Fennell et al. reported an extremely aggressive subset (20.8%) of BRAF^V600E^-mutant CRCs that is characterized by co-existing *APC* truncations [83]. Poor survival was recapitulated in *Apc*^min/+^ mice, in which BRAF^V600E^ accelerated disease progression by promoting massive polyp load. The underlying mechanisms and the behavior of *Braf*^*V600E*^*/Apc* double-mutant tumor cells were not investigated by Fennell et al., but our organoid data support their concept that BRAF^V600E^ and APC truncations induce an overt proliferative phenotype [83]. Despite the critical contribution of *Apc* deficiency to the fitness of BRAF^V600E^-expressing organoids, we observed that their growth and survival were still dependent on MEK activity. Remarkably, p53^R172H^ added a clear survival benefit to *Apc*-deficient BRAF^V600E^ expressing organoids exposed to the relatively high trametinib concentration of 100 nM. This indicates that p53^R172H^ confers drug resistance in this setting and represents important information for the design of rational combination therapies. The elucidation of the underlying mechanisms represents an area for further investigation.

In summary, our multistep reconstruction of CRC carcinogenesis (Fig. 7) provides new insights into frequently co-existing mutations of BRAF^V600E^ in human CRC and offers the opportunity to obtain mechanistic insights into emerging biomarkers and prognostic signatures. Furthermore, this genetically well-defined cross-species approach could help to identify metastasis and drug-resistance promoters. This could generate novel therapeutic concepts for an aggressive CRC subtype that poorly responds to chemo- and immunotherapy and requires the rational combination of various targeted therapy compounds for disease stabilization.

## Materials and methods

### Animals and organoid isolation

*Villin::CreER*^*T2*^ transgenic animals [27] were intercrossed with mice carrying conditional *Braf*^*floxV600E/+*^ [28] and/or *Trp53*^*LSL-R172H/+*^ (Olive et al. [29]) knock-in alleles. The conditional *APC*^*flox*^ allele [72] was kindly provided by Andreas Hecht (IMMZ). All lines were maintained on a C57Bl/6N background. Mice were kept under specific pathogen-free (SPF) conditions in the animal facility of the University Medical Center Freiburg according to institutional guidelines. Animals received standard diet and water ad libitum. Tissue was isolated from sacrificed mice in accordance with the German law for animal protection, and was approved by the government commission for animal protection and the local ethics committee (X-15/09H; X-18/06C; X-19/05C). Organoids were generated and propagated as described previously with minor modifications [21, 22]. A detailed description of this is provided in Supplementary Methods. CHIR-99021 and trametinib (GSK1120212) were purchased from Cayman Chemical and Selleck chemicals, respectively, and dissolved in DMSO.

### Colony-forming assay

COL organoids were disrupted into single cells by Accutase treatment for 10 min at 37 °C. Single cells were seeded in 50 µl Matrigel supplemented with 10 µM Y27632 onto a pre-warmed 24-well culture dish, and oncogene expression was subsequently induced with 3 µM 4-HT. After 4 days, bright-field images were taken, and the formed crypts measured.

### Anchorage-independent growth assay

Oncogene expression in COL organoids was induced with 3 µm 4-HT for 24 h. After 2 days, organoids were disrupted into single cells by Accutase for 10 min at 37 °C and put on PolyHEMA (5 mg/ml)-coated culture dishes. They were cultured for 6 days, while fresh crypt culture medium supplemented with growth factors was added on day 2 and 5, before bright-field images were taken and formed cell clusters measured.

### Invasion assay

Invasion assay was performed as described before with minor modifications [25]. COL organoids were disrupted into single cells by Accutase treatment for 10 min at 37 °C. Afterwards, single cells were seeded in 50 µl diluted (50%) Matrigel supplemented with 10 µM Y27632 onto a pre-cooled 24-well culture dish, and left at room temperature for 10 min. Subsequently, oncogene expression was induced with 3 µM 4-HT for 24 h. At day 7 bright-field images were taken, and organoids attached to the plastic surface were counted.

### Western blot analyses

Organoids were released from Matrigel by incubation with Cell Recovery Solution (BD Biosciences) at 4 °C for 30 min to 1 h, washed with PBS and pelleted, before they were lysed with RIPA lysis buffer. Protein concentration was determined using the BCA Protein Assay Kit (Thermo Fisher Scientific), and equal amounts were loaded on 10% SDS-PAGE. Standard western blot analysis was performed using the following primary antibodies against: AMACR (2A10) (1:1000, #3207, Cell Signaling), BRAF F7 (1:1000, sc-5284, Santa Cruz), BRAF^V600E^ (VE1) (1:100, kindly provided by Prof. A. v. Deimling and 1:750, ab228461, Abcam), CAV1 (D46G3) (1:1000, #3267, Cell Signaling), cleaved Caspase-3 (1:1000, #9661, Cell Signaling), CTSE (1:1000, ab36996, Abcam), DUSP6 (1:1000, LS-B5975, LifeSpan BioSciences), ECAD (1:1000, 610181, BD Biosciences), EPHA2 (1:1000, #6997, Cell Signaling), c-Fos (K-25) (1:1000, sc-253, Santa Cruz), GAPDH (1:2000, ab9489, Abcam), HSP90 (1:1000, #4874, Cell Signaling), MEK1/2 (1:1000, #9122, Cell Signaling), MYOF (D-11) (1:1000, sc-376879, Santa Cruz), NR2E3 (1:1000, sc-374513, Santa Cruz), OLFM4 (D6Y5A) (1:1000, #39141, Cell Signaling), Phospho-c-Fos (Ser32) (D82C12) (1:1000, #5348, Cell Signaling), Phospho-FRA1 (Ser265) (D22B1) (1:1000, #5841, Cell Signaling), Phospho-MEK1/2 (pS217/221) (1:1000, #9121, Cell Signaling), Phospho-P44/42 MAPK (Erk1/2) (Thr202/Tyr204) (1:2000, #9101, Cell Signaling), P44/42 MAPK (Erk1/2) (1:2000, #9102, Cell Signaling), 14–3–3 (1:1000, sc-1657, Santa Cruz), Vinculin (1:1000, #4650, Cell Signaling). HRP-conjugated anti-mouse or -rabbit secondary antibodies (800 µg/ml, Thermo Fisher Scientific) were used. Signals were detected using a Fusion Solo chemiluminescence reader, and quantified using the FusionCapt Advanced Software (VILBER LOURMAT).

### Immunofluorescence of FFPE sections

Organoids were fixed in-well with 4% paraformaldehyde (PFA) for 30 min and incubated with 70% ethanol for 1 h at room temperature. Afterwards, crypts were embedded into 2% agarose, dehydrated via a graded ethanol series, embedded into paraffin and sectioned at 5 µm. The sections were processed and stained using standard methods with the following primary antibodies against:

CTSE (1:100, ab36996, Abcam), ECAD (1:200, 610181, BD Biosciences), ITGB1 (1:150, 610467, BD Biosceiences), KI-67 (1:400, #9129, Cell Signaling), LYSC (1:50, sc-27958, Santa Cruz), PKCζ (1:300, sc-216, Santa Cruz), MUC2 (1:50, sc-15334, Santa Cruz). Secondary antibodies were: Alexa Fluor® 488/546 goat anti-rabbit/mouse, Alexa Fluor® 488 donkey anti-goat, Cy3® goat anti-mouse (all 1:200, Invitrogen). Finally, sections were mounted with ProLong^TM^ Gold Antifade with DAPI. Images were taken with the Zeiss AxioObserver Z1 plus ApoTome 2 with an AxioCam MR.

### Quantification and statistical analysis

Quantified data are presented as means ± SD. Statistical significance was analyzed using two-tailed paired or unpaired *t* test, as well as one-way ANOVA (corrected for multiple comparison by Bonferroni) as stated in the figure legends. Differences of *P ≤ 0.05, **P ≤ 0.01, ***P ≤ 0.001 were considered as statistically significant.

## Supplementary information

Supplementary Materials and data

Video S1

Video S2

Video S3

## References

[1] Dienstmann R, Vermeulen L, Guinney J, Kopetz S, Tejpar S, Tabernero J (2017). Consensus molecular subtypes and the evolution of precision medicine in colorectal cancer. Nat Rev Cancer.

[2] Jackstadt R, Sansom OJ (2016). Mouse models of intestinal cancer. J Pathol.

[3] Morkel M, Riemer P, Blaker H, Sers C (2015). Similar but different: distinct roles for KRAS and BRAF oncogenes in colorectal cancer development and therapy resistance. Oncotarget.

[4] Phipps AI, Limburg PJ, Baron JA, Burnett-Hartman AN, Weisenberger DJ, Laird PW (2015). Association between molecular subtypes of colorectal cancer and patient survival. Gastroenterology.

[5] Bond CE, Liu C, Kawamata F, McKeone DM, Fernando W, Jamieson S (2018). Oncogenic BRAF mutation induces DNA methylation changes in a murine model for human serrated colorectal neoplasia. Epigenetics.

[6] Rad R, Cadinanos J, Rad L, Varela I, Strong A, Kriegl L (2013). A genetic progression model of Braf-induced intestinal tumorigenesis reveals targets for therapeutic intervention. Cancer Cell.

[7] Sakamoto N, Feng Y, Stolfi C, Kurosu Y, Green M, Lin J, et al. BRAF(V600E) cooperates with CDX2 inactivation to promote serrated colorectal tumorigenesis. eLife 2017;6:e20331. 10.7554/eLife.20331 PMC5268782 28072391

[8] Carragher LA, Snell KR, Giblett SM, Aldridge VS, Patel B, Cook SJ (2010). V600EBraf induces gastrointestinal crypt senescence and promotes tumour progression through enhanced CpG methylation of p16INK4a. EMBO Mol Med.

[9] Riemer P, Sreekumar A, Reinke S, Rad R, Schafer R, Sers C (2015). Transgenic expression of oncogenic BRAF induces loss of stem cells in the mouse intestine, which is antagonized by beta-catenin activity. Oncogene.

[10] Herr R, Kohler M, Andrlova H, Weinberg F, Moller Y, Halbach S (2015). B-Raf inhibitors induce epithelial differentiation in BRAF-mutant colorectal cancer cells. Cancer Res.

[11] Tong K, Pellon-Cardenas O, Sirihorachai VR, Warder BN, Kothari OA, Perekatt AO (2017). Degree of tissue differentiation dictates susceptibility to BRAF-driven colorectal cancer. Cell Rep.

[12] Dawson H, Galvan JA, Helbling M, Muller DE, Karamitopoulou E, Koelzer VH (2014). Possible role of Cdx2 in the serrated pathway of colorectal cancer characterized by BRAF mutation, high-level CpG Island methylator phenotype and mismatch repair-deficiency. Int J Cancer.

[13] Clarke CN, Kopetz ES (2015). BRAF mutant colorectal cancer as a distinct subset of colorectal cancer: clinical characteristics, clinical behavior, and response to targeted therapies. J Gastrointest Oncol.

[14] Kastenhuber ER, Lowe SW (2017). Putting p53 in Context. Cell.

[15] Datta J, Smith JJ, Chatila WK, McAuliffe JC, Kandoth C, Vakiani E (2020). Coaltered Ras/B-raf and TP53 is associated with extremes of survivorship and distinct patterns of metastasis in patients with metastatic colorectal cancer. Clin Cancer Res.

[16] Kane AM, Fennell LJ, Liu C, Borowsky J, McKeone DM, Bond CE (2020). Alterations in signaling pathways that accompany spontaneous transition to malignancy in a mouse model of BRAF mutant microsatellite stable colorectal cancer. Neoplasia.

[17] Han T, Goswami S, Hu Y, Tang F, Zafra MP, Murphy C, et al. Lineage reversion drives WNT independence in intestinal cancer. Cancer Discov. 2020;CD-19-1536. 10.1158/2159-8290.CD-19-1536. Online ahead of print. 10.1158/2159-8290.CD-19-1536 PMC7541594 32546576

[18] Sekine S, Yamashita S, Yamada M, Hashimoto T, Ogawa R, Yoshida H (2020). Clinicopathological and molecular correlations in traditional serrated adenoma. J Gastroenterol.

[19] Hashimoto T, Ogawa R, Yoshida H, Taniguchi H, Kojima M, Saito Y (2019). Acquisition of WNT pathway gene alterations coincides with the transition from precursor polyps to traditional serrated adenomas. Am J Surg Pathol.

[20] Lannagan TRM, Lee YK, Wang T, Roper J, Bettington ML, Fennell L (2019). Genetic editing of colonic organoids provides a molecularly distinct and orthotopic preclinical model of serrated carcinogenesis. Gut.

[21] Sato T, Clevers H (2013). Primary mouse small intestinal epithelial cell cultures. Methods Mol Biol.

[22] Yui S, Nakamura T, Sato T, Nemoto Y, Mizutani T, Zheng X (2012). Functional engraftment of colon epithelium expanded in vitro from a single adult Lgr5(+) stem cell. Nat Med.

[23] de Sousa e Melo F, Kurtova AV, Harnoss JM, Kljavin N, Hoeck JD, Hung J (2017). A distinct role for Lgr5(+) stem cells in primary and metastatic colon cancer. Nature.

[24] Dow LE, O’Rourke KP, Simon J, Tschaharganeh DF, van Es JH, Clevers H (2015). Apc restoration promotes cellular differentiation and reestablishes crypt homeostasis in colorectal. Cancer Cell.

[25] Riemer P, Rydenfelt M, Marks M, van Eunen K, Thedieck K, Herrmann BG (2017). Oncogenic beta-catenin and PIK3CA instruct network states and cancer phenotypes in intestinal organoids. J Cell Biol.

[26] Laforest A, Aparicio T, Zaanan A, Silva FP, Didelot A, Desbeaux A (2014). ERBB2 gene as a potential therapeutic target in small bowel adenocarcinoma. Eur J Cancer.

[27] el Marjou F, Janssen KP, Chang BH, Li M, Hindie V, Chan L (2004). Tissue-specific and inducible Cre-mediated recombination in the gut epithelium. Genesis.

[28] Dankort D, Filenova E, Collado M, Serrano M, Jones K, McMahon M (2007). A new mouse model to explore the initiation, progression, and therapy of BRAFV600E-induced lung tumors. Genes Dev.

[29] Olive KP, Tuveson DA, Ruhe ZC, Yin B, Willis NA, Bronson RT (2004). Mutant p53 gain of function in two mouse models of Li-Fraumeni syndrome. Cell.

[30] Brandt R, Sell T, Luthen M, Uhlitz F, Klinger B, Riemer P (2019). Cell type-dependent differential activation of ERK by oncogenic KRAS in colon cancer and intestinal epithelium. Nat Commun.

[31] Herr R, Halbach S, Heizmann M, Busch H, Boerries M, Brummer T (2018). BRAF inhibition upregulates a variety of receptor tyrosine kinases and their downstream effector Gab2 in colorectal cancer cell lines. Oncogene.

[32] Rademaker G, Costanza B, Bellier J, Herfs M, Peiffer R, Agirman F (2019). Human colon cancer cells highly express myoferlin to maintain a fit mitochondrial network and escape p53-driven apoptosis. Oncogenesis.

[33] Wang D, Zhang J, Li Z, Han J, Gao Y, Chen M, et al. Upregulation of fibroblast growth factor 19 is associated with the initiation of colorectal adenoma. Dig Dis. 2018;37:214–25. 10.1159/000494454 30517925

[34] Liu QZ, Gao XH, Chang WJ, Gong HF, Fu CG, Zhang W (2015). Expression of ITGB1 predicts prognosis in colorectal cancer: a large prospective study based on tissue microarray. Int J Clin Exp Pathol.

[35] Caruso M, Moore J, Goodall GJ, Thomas M, Phillis S, Tyskin A (2009). Over-expression of cathepsin E and trefoil factor 1 in sessile serrated adenomas of the colorectum identified by gene expression analysis. Virchows Arch.

[36] Delker DA, McGettigan BM, Kanth P, Pop S, Neklason DW, Bronner MP (2014). RNA sequencing of sessile serrated colon polyps identifies differentially expressed genes and immunohistochemical markers. PLoS ONE.

[37] Sentani K, Sakamoto N, Shimamoto F, Anami K, Oue N, Yasui W (2013). Expression of olfactomedin 4 and claudin-18 in serrated neoplasia of the colorectum: a characteristic pattern is associated with sessile serrated lesion. Histopathology.

[38] Merlos-Suarez A, Barriga FM, Jung P, Iglesias M, Cespedes MV, Rossell D (2011). The intestinal stem cell signature identifies colorectal cancer stem cells and predicts disease relapse. Cell Stem Cell.

[39] Munoz J, Stange DE, Schepers AG, van de Wetering M, Koo BK, Itzkovitz S (2012). The Lgr5 intestinal stem cell signature: robust expression of proposed quiescent ‘+4’ cell markers. EMBO J.

[40] Boudreau F, Rings EH, van Wering HM, Kim RK, Swain GP, Krasinski SD (2002). Hepatocyte nuclear factor-1 alpha, GATA-4, and caudal related homeodomain protein Cdx2 interact functionally to modulate intestinal gene transcription. Implication for the developmental regulation of the sucrase-isomaltase gene. J Biol Chem.

[41] Herr R, Brummer T (2015). BRAF inhibitors in colorectal cancer: toward a differentiation therapy?. Mol Cell Oncol.

[42] Lavoie H, Sahmi M, Maisonneuve P, Marullo SA, Thevakumaran N, Jin T (2018). MEK drives BRAF activation through allosteric control of KSR proteins. Nature.

[43] Dorard C, Vucak G, Baccarini M (2017). Deciphering the RAS/ERK pathway in vivo. Biochem Soc Trans.

[44] Silberg DG, Sullivan J, Kang E, Swain GP, Moffett J, Sund NJ (2002). Cdx2 ectopic expression induces gastric intestinal metaplasia in transgenic mice. Gastroenterol.

[45] Kawazoe T, Taniguchi K (2019). The Sprouty/Spred family as tumor suppressors: coming of age. Cancer Sci.

[46] Verzi MP, Shin H, Ho LL, Liu XS, Shivdasani RA (2011). Essential and redundant functions of caudal family proteins in activating adult intestinal genes. Mol Cell Biol.

[47] Matsuda M, Sentani K, Noguchi T, Hinoi T, Okajima M, Matsusaki K (2010). Immunohistochemical analysis of colorectal cancer with gastric phenotype: claudin-18 is associated with poor prognosis. Pathol Int.

[48] Mustata RC, Vasile G, Fernandez-Vallone V, Strollo S, Lefort A, Libert F (2013). Identification of Lgr5-independent spheroid-generating progenitors of the mouse fetal intestinal epithelium. Cell Rep.

[49] Popovici, Budinska E, Tejpar S, Weinrich S, Estrella H, Hodgson G (2012). Identification of a poor-prognosis BRAF-mutant-like population of patients with colon cancer. J Clin Oncol.

[50] Khanal T, Choi K, Leung YK, Wang J, Kim D, Janakiram V (2017). Loss of NR2E3 represses AHR by LSD1 reprogramming, is associated with poor prognosis in liver cancer. Sci Rep.

[51] Park YY, Kim K, Kim SB, Hennessy BT, Kim SM, Park ES (2012). Reconstruction of nuclear receptor network reveals that NR2E3 is a novel upstream regulator of ESR1 in breast cancer. EMBO Mol Med.

[52] Macrae M, Neve RM, Rodriguez-Viciana P, Haqq C, Yeh J, Chen C (2005). A conditional feedback loop regulates Ras activity through EphA2. Cancer Cell.

[53] Dunne PD, Dasgupta S, Blayney JK, McArt DG, Redmond KL, Weir JA (2016). EphA2 expression is a key driver of migration and invasion and a poor prognostic marker in colorectal cancer. Clin Cancer Res.

[54] Ireton RC, Chen J (2005). EphA2 receptor tyrosine kinase as a promising target for cancer therapeutics. Curr Cancer Drug Targets.

[55] Campos A, Burgos-Ravanal R, Gonzalez MF, Huilcaman R, Lobos Gonzalez L, Quest AFG. Cell intrinsic and extrinsic mechanisms of caveolin-1-enhanced metastasis. Biomolecules*.* 2019;9:314. 10.3390/biom9080314 PMC6723107 31362353

[56] Torrejon B, Cristobal I, Rojo F, Garcia-Foncillas J (2017). Caveolin-1 is markedly downregulated in patients with early-stage colorectal cancer. World J Surg.

[57] Bender FC, Reymond MA, Bron C, Quest AF (2000). Caveolin-1 levels are down-regulated in human colon tumors, and ectopic expression of caveolin-1 in colon carcinoma cell lines reduces cell tumorigenicity. Cancer Res.

[58] Yang G, Truong LD, Timme TL, Ren C, Wheeler TM, Park SH (1998). Elevated expression of caveolin is associated with prostate and breast cancer. Clin Cancer Res.

[59] Aguirre-Portoles C, Feliu J, Reglero G, Ramirez de Molina A (2018). ABCA1 overexpression worsens colorectal cancer prognosis by facilitating tumour growth and caveolin-1-dependent invasiveness, and these effects can be ameliorated using the BET inhibitor apabetalone. Mol Oncol.

[60] Kitowska A, Wesserling M, Seroczynska B, Szutowicz A, Ronowska A, Peksa R (2015). Differentiation of high-risk stage I and II colon tumors based on evaluation of CAV1 gene expression. J Surg Oncol.

[61] Mi L, Zhu F, Yang X, Lu J, Zheng Y, Zhao Q (2017). The metastatic suppressor NDRG1 inhibits EMT, migration and invasion through interaction and promotion of caveolin-1 ubiquitylation in human colorectal cancer cells. Oncogene.

[62] Sotiriou C, Wirapati P, Loi S, Harris A, Fox S, Smeds J (2006). Gene expression profiling in breast cancer: understanding the molecular basis of histologic grade to improve prognosis. J Natl Cancer Inst.

[63] Wong DJ, Liu H, Ridky TW, Cassarino D, Segal E, Chang HY (2008). Module map of stem cell genes guides creation of epithelial cancer stem cells. Cell Stem Cell.

[64] Paoli P, Giannoni E, Chiarugi P (2013). Anoikis molecular pathways and its role in cancer progression. Biochim Biophys Acta.

[65] Tan BS, Tiong KH, Choo HL, Chung FF, Hii LW, Tan SH (2015). Mutant p53-R273H mediates cancer cell survival and anoikis resistance through AKT-dependent suppression of BCL2-modifying factor (BMF). Cell Death Dis.

[66] Li S, Chen Y, Zhang Y, Jiang X, Jiang Y, Qin X (2019). Shear stress promotes anoikis resistance of cancer cells via caveolin-1-dependent extrinsic and intrinsic apoptotic pathways. J Cell Physiol.

[67] Chanvorachote P, Pongrakhananon V, Halim H (2015). Caveolin-1 regulates metastatic behaviors of anoikis resistant lung cancer cells. Mol Cell Biochem.

[68] Halim H, Chanvorachote P (2012). Long-term hydrogen peroxide exposure potentiates anoikis resistance and anchorage-independent growth in lung carcinoma cells. Cell Biol Int.

[69] Rodrigues NR, Rowan A, Smith ME, Kerr IB, Bodmer WF, Gannon JV (1990). p53 mutations in colorectal cancer. Proc Natl Acad Sci USA.

[70] Fearon ER, Vogelstein B (1990). A genetic model for colorectal tumorigenesis. Cell.

[71] Duda P, Akula SM, Abrams SL, Steelman LS, Martelli AM, Cocco L, et al. Targeting GSK3 and associated signaling pathways involved in cancer. Cells. 2020;9:1110. 10.3390/cells9051110 PMC7290852 32365809

[72] Shibata H, Toyama K, Shioya H, Ito M, Hirota M, Hasegawa S (1997). Rapid colorectal adenoma formation initiated by conditional targeting of the Apc gene. Science.

[73] Muzumdar MD, Tasic B, Miyamichi K, Li L, Luo L (2007). A global double-fluorescent Cre reporter mouse. Genesis.

[74] Sundar R, Hong DS, Kopetz S, Yap TA (2017). Targeting BRAF-mutant colorectal cancer: progress in combination strategies. Cancer Discov.

[75] Kidger AM, Keyse SM (2016). The regulation of oncogenic Ras/ERK signalling by dual-specificity mitogen activated protein kinase phosphatases (MKPs). Semin Cell Dev Biol.

[76] Murphy LO, Smith S, Chen RH, Fingar DC, Blenis J (2002). Molecular interpretation of ERK signal duration by immediate early gene products. Nat Cell Biol.

[77] Gillies TE, Pargett M, Minguet M, Davies AE, Albeck JG (2017). Linear integration of ERK activity predominates over persistence detection in Fra-1 regulation. Cell Syst.

[78] Zhou X, Hao Q, Lu H (2019). Mutant p53 in cancer therapy-the barrier or the path. J Mol Cell Biol.

[79] Tao Y, Kang B, Petkovich DA, Bhandari YR, In J, Stein-O’Brien G (2019). Aging-like spontaneous epigenetic silencing facilitates wnt activation, stemness, and Braf(V600E)-induced tumorigenesis. Cancer Cell.

[80] Wang Y, Xu X, Maglic D, Dill MT, Mojumdar K, Ng PK (2018). Comprehensive molecular characterization of the hippo signaling pathway in cancer. Cell Rep.

[81] Kawasaki K, Fujii M, Sugimoto S, Ishikawa K, Matano M, Ohta Y (2020). Chromosome engineering of human colon-derived organoids to develop a model of traditional serrated adenoma. Gastroenterology.

[82] Bond CE, Whitehall VLJ (2018). How the BRAF V600E mutation defines a distinct subgroup of colorectal cancer: molecular and clinical implications. Gastroenterol Res Pr.

[83] Fennell LJ, Kane A, Liu C, McKeone D, Fernando W, Su C, et al. APC mutation marks an aggressive subtype of BRAF mutant colorectal cancers. Cancers. 2020;12:1171. 10.3390/cancers12051171 PMC7281581 32384699

