# Supplementary material for: BRAFV600E drives dedifferentiation in small intestinal and colonic organoids and cooperates with mutant p53 and Apc loss in transformation
Source: Oncogene. 2020 Aug 13;39(38):6053–70. doi: 10.1038/s41388-020-01414-9 (PMC7498370; doi:10.1038/s41388-020-01414-9)

Supplementary Materials for

BRAF^{V600E} drives dedifferentiation in small intestinal and colonic organoids and cooperates with mutant p53 and Apc loss in transformation

Nadine Reischmann, Geoffroy Andrieux, Ricarda Griffin, Thomas Reinheckel, Melanie Boerries and Tilman Brummer*

*Corresponding author: tilman.brummer@mol-med.uni-freiburg.de

This PDF file includes:

1. Supplementary Methods
2. Supplementary Figure legends
3. Supplementary Table 1
4. Supplementary Table 2
5. Supplementary References
6. Supplementary Figure 1
7. Supplementary Figure 2
8. Supplementary Figure 3
9. Supplementary Figure 4
10. Supplementary Figure 5
11. Supplementary Figure 6
12. Supplementary Figure 7
13. Supplementary Figure 8
14. Supplementary Figure 9
15. Supplementary Figure 10

1. Supplementary Methods

Establishment of organoids

The proximal small intestine or colon were isolated, flushed with ice-cold PBS and opened longitudinally. In the small intestine, the villi were scraped off using a glass slide. Afterwards, the intestine was put into a falcon tube containing 20 ml ice-cold PBS and vortexed for 5-10 sec. This washing was repeated 5-10 times until the supernatant was almost clear. The intestine was subsequently incubated in 30 ml ice-cold crypt isolation buffer (small intestine: 2 mM EDTA in PBS for 30 min; colon: 15 mM EDTA in PBS for 1 h) at 4 °C on a rotating mixer. The intestine was placed into 20 ml ice-cold PBS, inverted 10 times and then transferred into a fresh falcon tube with 10 ml ice-cold PBS and shaken about 10 times (= supernatant 1). Subsequently, the intestine was transferred into fresh 10 ml ice-cold PBS and shaken again. The crypt releasing procedure was repeated 10 times and each supernatant was inspected under the microscope. The crypt containing fractions were pooled, centrifuged at 100 x g for 10 min and the supernatant was discarded. Afterwards, the crypt pellet was resuspended with 10 ml Advanced DMEM/F12 containing 2 mM L-glutamine, 10 mM HEPES, 100 U/ml penicillin and 100 µg/ml streptomycin (all from PAN-Biotech GmbH), passed through a 70-µm cell strainer and collected in a 1% BSA-coated falcon tube. The crypts were spun down at 300 x g for 5 min, resuspended with Matrigel (Corning) and seeded in 50 µl onto a pre-warmed 24-well culture dish. After the Matrigel had solidified, the crypts were overlaid with 500 µl complete crypt culture medium (Advanced DMEM/F12 with 2 mM L-glutamine, 10 mM HEPES, 100 U/ml penicillin, 100 µg/ml streptomycin, 1xN2, 1xB27 (both from Thermo Fisher), 81.5 mg/ml N-Acetylcystein (Sigma-Aldrich)), supplemented with 100 ng/ml murine recombinant Noggin, 500 ng/ml human recombinant R-Spondin1, 50 ng/ml murine recombinant EGF (all from Peprotech) and 10 µM Rho kinase inhibitor Y27632 (TOCRIS). For colonic crypts 50 ng/ml recombinant murine Wnt3a (Peprotech) and 2.5 µM GSK3i CHIR99021 (Cayman Chemical) were added.

Medium was changed every 3-4 days and crypts were passaged once a week to remove dead cell debris.

Global induction of Cre recombinase was achieved by treatment of the organoids with 1 µM (for small intestinal crypts) and 3 µM (for colonic crypts) 4-HT ((Z)-4-hydroxytamoxifen (Sigma-Aldrich)), respectively, for 24 h.

Cre mediated recombination of *Braf*^{floxV600E} and *Apc*^{flox} was assessed by genomic PCR using primers that were described previously [1, 2] and Cre mediated recombination of *Trp53*$^{LSL\text{-}R172H/+}$ was assessed using the following primers: 5'-AGCCTTAGACATAACACACGAACT-3', 5'-CTTGGAGACATAGCCACACTG-3', 5'-GCCACCATGGCTTGAGTAA-3'.

MTT staining of organoids

Organoids were incubated with 500 µg/ml MTT solution diluted in DMEM/F12 for 2 h at 37 °C.

RNA-sequencing

For the RNA-Seq of small intestine (SI) and colon (COL), RNA was extracted from freshly isolated SI and COL crypts of four (two female and male, respectively) mice. For the RNA-Seq of organoids, the organoid cultures of three donor mice (*Trp53*$^{LSL\text{-}R172H/+}$*;Villin::CreER*^{T2}*,* *Braf*$^{floxV600E/+}$*;Villin::CreER*^{T2}*,* *Braf*$^{floxV600E/+}$*;Trp53*$^{LSL\text{-}R172H/+}$*;Villin::CreER*^{T2}) were induced with 4-HT (0.5 µM for SI organoids, 3.5 µM for COL organoids) in duplicates for 24 h. SI crypt RNA was isolated after 8 days and COL crypt RNA was isolated after 3 days. Isolated RNA, from 24 organoid- and 8 crypt samples, was sequenced on an Illumina HiSeq4000. Paired-end reads were filtered to remove bad quality reads and adapter sequences using Trimmomatic [3]. The selected reads were aligned to the mouse reference genome mm10 and read count per gene was quantified with STAR aligner [4]. A linear model-based algorithm, limma [5], was used to calculate the differentially regulated genes on the following contrasts: *Braf*^{V600E} on vs. off, *Trp53*^{R172H} on vs. off, *Braf*^{V600E} and *Trp53*^{R172H} on vs. off for colonic and small intestinal organoids separately. For wildtype crypts we compared the small intestine vs. colon replicates. The same significant threshold was used for all comparisons, i.e. adjusted p-value (Benjamini-Hochberg) below 0.05. The gene-set enrichment analysis was performed using the fgsea R package [6], where genes were ranked according to the on vs. off log2 fold-change and tested against the MSigDB [7] gene-sets (Hallmark, chemical and genetic perturbations, Biocarta, KEGG, PID, Reactome, miRNA, transcription factor targets, cancer modules, Gene Ontology, oncogenic signature and immunologic signature) as well as the SSAs UP and DOWN signature lists (GSE45270), the *Cdx1/Cdx2* double knockout signature (GSE24633) and the mouse fetal intestinal spheroid signature [8].

Data availability

RNA-Seq data were uploaded to Gene Expression Omnibus database.
To review GEO accession GSE132551, go to:
https://www.ncbi.nlm.nih.gov/geo/query/acc.cgi?acc=GSE132551
Enter token avgfcaecltkpbmf into the box.
To review GEO accession GSE132546:
Go to https://www.ncbi.nlm.nih.gov/geo/query/acc.cgi?acc=GSE132546
Enter token mlyzwysyltuhfoj into the box

TCGA data

RNA-Seq raw counts were downloaded from TCGA using TCGAbiolinks R package [9]. $BRAF^{V600E}$ mutant colorectal adenocarcinoma were selected (n=54), as well as healthy colon samples (n=41). Counts were converted into log2 count per million (CPM) for further analysis. GSEA analysis was performed on the average log2 foldchange between COAD and healthy colon using fgsea R package.

2. Supplementary Figure legends

Fig. S1. Confirmation and effects of Villin::CreER^{T2}-activated recombination within SI and COL organoids.

(**a**) Schematic models of Villin::CreER^{T2}-activated recombination. Following 4-HT induction, BRAF^{V600E} or dominant negative acting p53^{R172H} are expressed under the control of their own promoters.

(**b**) Confirmation of successful recombination within 4-HT-treated organoids by genomic PCR. DNA of donor mice serve as controls and EtOH serves as vehicle control for 4-HT.

(**c**) Western blot (WB) of SI crypts. Treatment with 4-HT induces expression of BRAF^{V600E} and downstream signaling, indicated by the phosphorylation of MEK and ERK and increased expression of their target gene DUSP6. The tight control of CreER^{T2} is further demonstrated by a BRAF^{V600E} specific antibody [10], showing immunoreactivity only in lysates of 4-HT-treated organoid cultures.

(**d**) Time lapse images of disintegrating organoids following 4-HT induction. The Paneth cells (white arrows) disappear within SI organoids after oncogene expression.

(**e**) Quantification of organoid disintegration time after 4-HT induction. Data are presented as mean ± SD. $^{**}P \leq 0.01$; $^{****}P \leq 0.0001$ (one-way ANOVA, corrected for multiple comparison by Bonferroni) ($n \geq 3$ independent 4-HT inductions).

(**f**) BRAF^{V600E}-mutant COL organoids were induced with 3 µM 4-HT or left untreated. After 4 days, organoids were stained with 1.5 µM DRAQ7 for 10 min to visualize dead cells. Fluorescent and bright-field images are shown.

(**g**) WB of COL crypt lysates three days following 4-HT induction. BRAF^{V600E} and BRAF^{V600E}/p53^{R172H} samples show increased presence of the large fragment (17/19 kDA) of activated CASPASE-3. Detection of 14-3-3 serves as loading control.

Figure information: In (d,f), scale bars: 50 µm.

Fig. S2. BRAF^{V600E} expression leads to profound changes in organoid organization.

(**a**) Induction of *Trp53*$^{R172H/+}$ neither leads to disintegration of SI nor COL organoids, and Paneth cells (white arrow) remain visible within *Trp53*$^{R172H/+}$ mutant SI crypts. Scale bar: 50 µm.

(**b**) Control SI organoids, which are unable to express $BRAF^{V600E}$ due to the lack of floxed alleles or cre recombinase, do not show any morphological changes after treatment with the indicated 4-HT concentrations. Scale bars: 50/100 µm.

(**c-e**) Side-by-side comparison of epithelial features in SI and COL organoids expressing $BRAF^{V600E}$ and/or $p53^{R172H}$. Different time points of *Trp53*$^{R172H/+}$ mutant COL organoids are shown to illustrate that $p53^{R172H}$ by itself does not disturb epithelial organization, neither at an early nor at a later time point following oncogene induction.

(**c**) KI-67 and E-Cadherin immunofluorescence (IF) staining of formalin-fixed paraffin-embedded (FFPE) organoid sections. Clusters positive for the S-phase marker KI-67 were confined to the stem cell niches of control organoids and those expressing $p53^{R172H}$. In contrast, organoids expressing $BRAF^{V600E}$, either singly or in combination with $p53^{R172H}$, displayed extended stretches of KI-67 positive cells that often occurred outside of morphologically defined crypt bases. Note that the organization of the single-layered polarized epithelium appeared disturbed and the nuclei no longer exhibited their basal deposition.

(**d**) The establishment of an apico-basal-polarity axis represents a defining moment in epithelial organogenesis and for the sealing of epithelia by the proper deposition of tight junctions. Therefore, localization of PKCζ, a widely used marker for the identification of the apical surface of epithelia [11], was assessed by IF. Note that PKCζ is deposited at the luminal (apical) plasma membranes in control and $p53^{R172H}$ expressing SI and COL organoids, while its typical localization is absent in the presence of $BRAF^{V600E}$.

(**e**) Choleratoxin (CTX; 0.1 µg/ml) was used to assess the integrity of tight junctions in spheroid culture, as this toxin triggers ion mediated water efflux into the spheroid lumen [11]. Only if tight junctions are sealed, the lumen will expand in response to CTX. In line with our previous data showing that $BRAF^{V600E}$ impairs the seal of tight junctions in human CRC cell line spheroids [10, 12], we demonstrate that CTX triggers luminal expansion in control and $p53^{R172H}$ expressing SI and COL organoids but not in those expressing $BRAF^{V600E}$. This indicates that $BRAF^{V600E}$ impairs epithelial sealing.

(**f**) IF staining for Lysozyme C in SI organoids. The niche of SI stem cells is organized by Paneth cells, which supply the crypt base with growth factors and can be visualized by lysozyme expression [13]. In agreement with the loss of

morphologically defined crypts and the disappearance of Paneth cells in bright-field micrographs (**Fig. S1d**), lysozyme positive Paneth cells were almost completely absent in organoids expressing $BRAF^{V600E}$. This suggests that Paneth cells are lost upon $BRAF^{V600E}$ expression, which agrees with previous findings that ERK promotes Goblet cell generation at the expense of Paneth cells [14].
Figure information: In (c-f), scale bars: 50 µm.

Fig. S3. Validation of RNA-Seq analysis.
(**a**) PCA of RNA-Seq of SI (circles) and COL (triangles) crypts. Color code represents the different conditions where "off" corresponds to EtOH-treated control organoids and "on" corresponds to 4-HT induced organoids.
(**b**) IF staining of FFPE organoid sections indicates increased membranous β1-Integrin (ITGB1) within $BRAF^{V600E}$ and double-mutant organoids.
(**c**) CTSE IF staining of FFPE organoid sections. $BRAF^{V600E}$ mutant SI and COL organoids show increased Cathepsin E production, which is even more enhanced by the addition of $p53^{R172H}$.
(**d**) WB analyses and densitometric quantification of NR2E3 and Ephrin type-A receptor 2 (EPHA2) expression in COL organoids. Protein expression was normalized to corresponding loading control and EtOH-treated control organoids. N=3 independent biological replicates.
(**e**) Densitometric quantification of the WB analyses of CTSE and CAV1 shown in **Fig. 1c** and four and two additional biological replicates, respectively. Protein expression was normalized to corresponding loading control and EtOH-treated control organoids.
Figure information: In (b,c), scale bars: 50 µm. In (d,e), data are presented as mean ± SD. $*P \leq 0.05$; $**P \leq 0.01$; $***P \leq 0.001$; $****P \leq 0.0001$ (one-way ANOVA, corrected for multiple comparison by Bonferroni) ($n \geq 3$ independent experiments).

Fig. S4. $BRAF^{V600E}$ mutant organoids resemble the gene signature of SSAs.
$Braf^{V600E/+}$ and double-mutant COL organoids show enrichment and depletion of genes that are expressed at high and low levels (top 500), respectively, in SSAs when compared to tubular adenomas (TAs) from familiar adenomatous polyposis patients, who harbor an *APC* germline mutation (GSE45270).

Fig. S5. GSEA of the delta log2 fold-changes of the $BRAF^{V600E}$-only and double-mutant organoids.

Barplots of the top 50 (sorted by NES) UP (left panel) and DOWN (right panel) regulated MSigDB gene sets. Enrichment was performed on the calculated delta log2 fold-change between the double-mutant and $BRAF^{V600E}$-only in the SI (**a**) and COL (**b**) organoids.

Fig. S6. Analysis of the expression of EMT marker genes in *Braf*$^{V600E/+}$ (VE) single and *Braf*$^{V600E/+}$,*Trp53*$^{R172H/+}$ (VE,p53) double mutant COL organoids. Heatmap of the log2 fold-changes (4-HT induced vs. non-induced, color coded) of the genes listed in the EMT hallmark gene set.

Fig. S7. *Braf*$^{V600E/+}$,*Trp53*$^{R172H/+}$-mutant COL organoids show enhanced invasive morphology.

Bright-field images at a higher magnification of the invaded organoids shown in Fig. 4 G/H. Double-mutant COL organoids present with faster and increased attachment to the plastic surface and with pseudopodia formation (marked with white arrows). Scale bars: 50 µm.

Fig. S8. Analysis of 4-HT induced APC deficiency in COL organoids.

(**a**) Oncogene expression was induced and COL organoids were simultaneously treated with 7 µM of GSK inhibitor (GSK3i). Bright-field images are shown 4 days after oncogene induction. Scale bars: 50 µm.

(**b**) Confirmation of successful recombination of the *Apc*^{flox} allel within 4-HT-treated COL organoids by genomic PCR. DNA of donor mouse serves as control and EtOH serves as vehicle control for 4-HT.

(**c**) WB of COL organoids demonstrating ERK pathway activation and OLFM4 preservation within APC deficient organoids

(**d**) Schematic model of the *Rosa26::mTOM/mGFP* reporter allele, which produces a membrane-tethered tdTomato (mTOM) protein and a membrane-bound green fluorescent protein (mGFP) prior to and following Cre mediated recombination, respectively.

(**e**) Bright-field and fluorescent microscopy images of COL organoids treated with 0.1 µg/ml CTX for 24 hours. *Braf*$^{V600E/+}$,*Apc*$^{\Delta/\Delta}$ mutant organoids do not show CTX-

induced luminal expansion, indicating impaired tight junction formation (see **Fig. S2e** for more details). Scale bars: 50 µm.

Fig. S9. WB analysis of trametinib-treated COL organoids.

(**a**) COL organoids with the indicated genotypes (numbers 1-3 refer to different triple-mutant donor mice) were induced with 3 µM 4-HT. They were treated with 25 nM trametinib for 24 h before they were lysed and analyzed by WB with the indicated antibodies. Detection of GAPDH or Vinculin serves as loading control.

(**b**) Densitometric quantification of the WB analyses shown in (**a**) and two additional biological replicates. Protein expression was normalized to GAPDH/ Vinculin and DMSO-treated double-mutant organoids. Data are presented as mean ± SD. **P ≤ 0.01 (one-way and two-way ANOVA, respectively, corrected for multiple comparison by Bonferroni) (n=3 independent experiments).

Fig. S10. BRAF^{V600E} upregulates YAP/TAZ targets in murine organoids.

Analysis of Hippo pathway activity by YAP/TAZ transcriptional targets identified by Wang and colleagues in SI and COL organoids. Color code represents the log2 fold-change (induced vs. non-induced). *Braf$^{V600E/+}$* (VE); *Braf$^{V600E/+}$,Trp53$^{R172H/+}$* (VE,p53).

Videos S1,2.

Time lapse videos (every 4h) of SI (**Video S1**) and COL (**Video S2**) organoids at days 1-6 after oncogene induction. Note the fast proliferation of mutant organoids, especially of COL organoids in video supplement 2. Scale bars: 250 µm.

Video S3.

Time lapse videos (every 1 h) of COL organoids with the indicated genotypes at days 2-5 after oncogene induction. Scale bars: 250 µm.

3. Table S1. Summary of studies using $BRAF^{V600E}$ mutant SI and COL organoids.

Reference	Model	Time point of 4-HT (or dox) treatment	Culture conditions	Organoid type	Observation on organoids
Riemer et al., 2015	dox inducible *Braf*^{V600K} transgene knocked into *Rosa26* locus	*in vivo* (dox) via drinking water and *in vitro*	(Sato et al., 2011)	SI	no organoid survival after isolation of dox treated mice; cell death of dox treated organoids; loss of stem cell niche; rescue by BRAF and MEK inhibition or Wnt pathway activation
Fessler et al., 2016	introduction of *BRAF*^{V600E} in human COL organoids by homologous recombination	Not applicable	(Prasetyanti et al., 2013; Sato et al., 2011)	COL	no disintegration observed
Tong et al., 2017	Villin-CreER^{T2} + *Braf*^{floxV600E} mice (Dankort et al., 2007)	*in vivo* via intraperitoneal injection	2.5% R-Spondin conditioned-media	SI	no organoid survival after isolation; loss of Paneth cells; reduction of *Cdx2* gene, loss of *SMAD4* or elevated WNT signaling rescue organoids
Sakamoto et al., 2017	Cdx2P-CreER^{T2} + *Braf*^{floxV600E} mice (Dankort et al., 2007)	*in vivo* via intraperitoneal injection	(Yui et al., 2012); 10% R-spondin2 (Bell et al., 2008), 10% Wnt3A (Barker et al., 2010) conditioned-media; 50 ng/ml mouse HGF; 10 µM Y-27632	COL	no disintegration observed
Bond et al., 2018	Villin-CreER^{T2} + *Braf*^{floxV600E} mice (Dankort et al., 2007)	1 µM *in vitro*	(Sato et al., 2011); Matrigel "sandwich"	COL	no disintegration observed
Lannagan et al., 2019	Villin-CreER^{T2} + *Braf*^{floxV600E} mice (Dankort et al., 2007)	1 µM *in vitro*	Matrigel "sandwich" (Onuma et al., 2013); 20% R-spondin2, 50% Wnt3A conditioned-media	COL	no disintegration observed
Tao et al., 2019	Lentiviral Cre delivery in organoids derived from *Braf*^{floxV600E} mice (Dankort et al., 2007)	Not applicable	(Weeber et al., 2015); 10% Noggin, 20% R-Spondin1, 50% Wnt3A conditioned-media	COL	cell death upon $BRAF^{V600E}$ induction in organoids at low passage number. Long-term cultured organoids are less sensitive to oncogene induction
Brandt et al., 2019	dox inducible *Braf*^{V600E} transgene knocked into *Rosa26* locus	*in vitro* (dox)	R-Spondin conditioned-media	SI	cell death upon $BRAF^{V600E}$ induction; epithelial disorganization; rescue of organoid death by MEK or ERK inhibitors

4. Table S2. Summary of selected $BRAF^{V600E}$ regulated genes that have been validated by WB or IHC and their relevance in human CRC and other cancers.

Gene	Response to $BRAF^{V600E}$ induction in murine organoids	Alteration in human colorectal and other cancers	Prognostic relevance
**AMACR**	downregulated	Strongly expressed in differentiated CRC, but weakly expressed in anaplastic CRC as assessed by IHC [30-32]. Low expression correlates with mucinous histology [33] and right-sided tumors [34].	Potential marker for tumor differentiation/neoplastic change and significant association with prognostic factors such as tumor grade, tumor stage and tumor location [30-32, 34].
**CAV1**	upregulated	Increased expression in high grade tumors with greater depth of invasiveness [35].	Potential tumor suppressor gene [36] and potential marker for selecting low- and high-stage CRC patients [35].
**CTSE**	upregulated	Upregulated, in particular in SSAs as shown by IHC and quantitative RT-PCR [37, 38].	Potential marker for SSA [37].
**EPHA2**	upregulated	High mRNA and protein expression are associated with poor overall survival in stage II/III CRC (shown using a CRC microarray dataset and IHC) [39]. High expression correlates with metastatic spread [40].	Poor prognostic marker in stage II/III CRC and EPHA2-targeted agents as potential treatment strategy [39].
**ITGB1**	upregulated	High expression in advanced tumors and liver metastases as shown by IHC. High expression correlates with shortened OS and DFS [41].	Potential biomarker for disease progression [41].
**MUC2**	upregulated	Increased expression of MUC2 in mucinous CRC. Significantly higher incidence of BRAF mutations in mucinous CRC [42, 43].	Mucinous CRCs often present at an advanced stage and correlate with poor survival [43, 44].
**MYOF**	upregulated	Overexpressed in human CRC as shown by IHC staining and TCGA gene expression data sets [45].	High expression is associated with lower survival in CRC patients [45].
**NR2E3**	downregulated	**No data for CRC so far.** Low expression in ERα-positive breast cancer patients associated with worse recurrence-free survival [46]. Low expression in liver tumors as assessed by IHC [47].	Potential tumor suppressor in breast and liver cancer [46, 47].
**OLFM4**	downregulated	IHC and proteomic approaches show upregulation of OLFM4 in adenoma and early CRC stages compared to normal epithelium. There is no difference between OLFM4 expression in stage III/IV CRC vs. normal tissue [48, 49].	Reduced OLFM4 (also known as hGC-1) expression as potential marker for poor differentiation and malignant progression of CRC [49].

5. Supplementary References

1 Dankort D, Filenova E, Collado M, Serrano M, Jones K, McMahon M. A new mouse model to explore the initiation, progression, and therapy of BRAFV600E-induced lung tumors. *Genes Dev* 2007; 21: 379-384.

2 Shibata H, Toyama K, Shioya H, Ito M, Hirota M, Hasegawa S *et al*. Rapid colorectal adenoma formation initiated by conditional targeting of the Apc gene. *Science* 1997; 278: 120-123.

3 Bolger AM, Lohse M, Usadel B. Trimmomatic: a flexible trimmer for Illumina sequence data. *Bioinformatics* 2014; 30: 2114-2120.

4 Dobin A, Davis CA, Schlesinger F, Drenkow J, Zaleski C, Jha S *et al*. STAR: ultrafast universal RNA-seq aligner. *Bioinformatics* 2013; 29: 15-21.

5 Ritchie ME, Phipson B, Wu D, Hu Y, Law CW, Shi W *et al*. limma powers differential expression analyses for RNA-sequencing and microarray studies. *Nucleic Acids Res* 2015; 43: e47.

6 Sergushichev AA. An algorithm for fast preranked gene set enrichment analysis using cumulative statistic calculation. *bioRxiv* 2019.

7 Liberzon A, Birger C, Thorvaldsdottir H, Ghandi M, Mesirov JP, Tamayo P. The Molecular Signatures Database (MSigDB) hallmark gene set collection. *Cell Syst* 2015; 1: 417-425.

8 Mustata RC, Vasile G, Fernandez-Vallone V, Strollo S, Lefort A, Libert F *et al*. Identification of Lgr5-independent spheroid-generating progenitors of the mouse fetal intestinal epithelium. *Cell Rep* 2013; 5: 421-432.

9 Colaprico A, Silva TC, Olsen C, Garofano L, Cava C, Garolini D *et al*. TCGAbiolinks: an R/Bioconductor package for integrative analysis of TCGA data. *Nucleic Acids Res* 2016; 44: e71.

10 Röring M, Herr R, Fiala GJ, Heilmann K, Braun S, Eisenhardt AE *et al*. Distinct requirement for an intact dimer interface in wild-type, V600E and kinase-dead B-Raf signalling. *EMBO J* 2012; 31: 2629-2647.

11 Jaffe AB, Kaji N, Durgan J, Hall A. Cdc42 controls spindle orientation to position the apical surface during epithelial morphogenesis. *J Cell Biol* 2008; 183: 625-633.

12 Herr R, Köhler M, Andrlová H, Weinberg F, Möller Y, Halbach S *et al*. B-Raf inhibitors induce epithelial differentiation in BRAF-mutant colorectal cancer cells. *Cancer Res* 2015; 75: 216-229.

13 Sato T, van Es JH, Snippert HJ, Stange DE, Vries RG, van den Born M *et al*. Paneth cells constitute the niche for Lgr5 stem cells in intestinal crypts. *Nature* 2011; 469: 415-418.

14 Heuberger J, Kosel F, Qi J, Grossmann KS, Rajewsky K, Birchmeier W. Shp2/MAPK signaling controls goblet/paneth cell fate decisions in the intestine. *Proc Natl Acad Sci U S A* 2014; 111: 3472-3477.

15 Riemer P, Sreekumar A, Reinke S, Rad R, Schafer R, Sers C *et al*. Transgenic expression of oncogenic BRAF induces loss of stem cells in the mouse intestine, which is antagonized by beta-catenin activity. *Oncogene* 2015; 34: 3164-3175.

16 Fessler E, Drost J, van Hooff SR, Linnekamp JF, Wang X, Jansen M *et al*. TGFbeta signaling directs serrated adenomas to the mesenchymal colorectal cancer subtype. *EMBO Mol Med* 2016; 8: 745-760.

17 Prasetyanti PR, Zimberlin CD, Bots M, Vermeulen L, Melo Fde S, Medema JP. Regulation of stem cell self-renewal and differentiation by Wnt and Notch are conserved throughout the adenoma-carcinoma sequence in the colon. *Mol Cancer* 2013; 12: 126.

18 Tong K, Pellon-Cardenas O, Sirihorachai VR, Warder BN, Kothari OA, Perekatt AO *et al*. Degree of Tissue Differentiation Dictates Susceptibility to BRAF-Driven Colorectal Cancer. *Cell Rep* 2017; 21: 3833-3845.

19 Sakamoto N, Feng Y, Stolfi C, Kurosu Y, Green M, Lin J *et al*. BRAF(V600E) cooperates with CDX2 inactivation to promote serrated colorectal tumorigenesis. *Elife* 2017; 6.

20 Yui S, Nakamura T, Sato T, Nemoto Y, Mizutani T, Zheng X *et al*. Functional engraftment of colon epithelium expanded in vitro from a single adult Lgr5(+) stem cell. *Nat Med* (In Vitro Research Support, Non-U.S. Gov't) 2012; 18: 618-623.

21 Bell SM, Schreiner CM, Wert SE, Mucenski ML, Scott WJ, Whitsett JA. R-spondin 2 is required for normal laryngeal-tracheal, lung and limb morphogenesis. *Development* 2008; 135: 1049-1058.

22 Barker N, Huch M, Kujala P, van de Wetering M, Snippert HJ, van Es JH *et al*. Lgr5(+ve) stem cells drive self-renewal in the stomach and build long-lived gastric units in vitro. *Cell Stem Cell* 2010; 6: 25-36.

23 Bond CE, Liu C, Kawamata F, McKeone DM, Fernando W, Jamieson S *et al*. Oncogenic BRAF mutation induces DNA methylation changes in a murine model for human serrated colorectal neoplasia. *Epigenetics* 2018; 13: 40-48.

24 Lannagan TRM, Lee YK, Wang T, Roper J, Bettington ML, Fennell L *et al*. Genetic editing of colonic organoids provides a molecularly distinct and orthotopic preclinical model of serrated carcinogenesis. *Gut* 2019; 68: 684-692.

25 Onuma K, Ochiai M, Orihashi K, Takahashi M, Imai T, Nakagama H *et al*. Genetic reconstitution of tumorigenesis in primary intestinal cells. *Proc Natl Acad Sci U S A* 2013; 110: 11127-11132.

26 Tao Y, Kang B, Petkovich DA, Bhandari YR, In J, Stein-O'Brien G *et al*. Aging-like Spontaneous Epigenetic Silencing Facilitates Wnt Activation, Stemness, and Braf(V600E)-Induced Tumorigenesis. *Cancer Cell* 2019; 35: 315-328 e316.

27 Weeber F, van de Wetering M, Hoogstraat M, Dijkstra KK, Krijgsman O, Kuilman T *et al*. Preserved genetic diversity in organoids cultured from biopsies of human colorectal cancer metastases. *Proc Natl Acad Sci U S A* 2015; 112: 13308-13311.

28 Brandt R, Sell T, Luthen M, Uhlitz F, Klinger B, Riemer P *et al*. Cell type-dependent differential activation of ERK by oncogenic KRAS in colon cancer and intestinal epithelium. *Nat Commun* 2019; 10: 2919.

29 Sato T, Stange DE, Ferrante M, Vries RG, Van Es JH, Van den Brink S *et al*. Long-term expansion of epithelial organoids from human colon, adenoma, adenocarcinoma, and Barrett's epithelium. *Gastroenterology* 2011; 141: 1762-1772.

30 Kuefer R, Varambally S, Zhou M, Lucas PC, Loeffler M, Wolter H *et al*. alpha-Methylacyl-CoA racemase: expression levels of this novel cancer biomarker depend on tumor differentiation. *Am J Pathol* 2002; 161: 841-848.

31 Shi X, Gong E, Wu X. Alpha-methylacyl-CoA racemase/P504S overexpression in colorectal carcinoma is correlated with tumor differentiation. *Appl Immunohistochem Mol Morphol* 2007; 15: 175-180.

32 Shukla N, Adhya AK, Rath J. Expression of Alpha - Methylacyl - Coenzyme A Racemase (AMACR) in Colorectal Neoplasia. *J Clin Diagn Res* 2017; 11: EC35-EC38.

33 Chen ZM, Ritter JH, Wang HL. Differential expression of alpha-methylacyl coenzyme A racemase in adenocarcinomas of the small and large intestines. *Am J Surg Pathol* 2005; 29: 890-896.

34 Marx A, Simon P, Simon R, Mirlacher M, Izbicki JR, Yekebas E *et al*. AMACR expression in colorectal cancer is associated with left-sided tumor localization. *Virchows Arch* 2008; 453: 243-248.

35 Kitowska A, Wesserling M, Seroczynska B, Szutowicz A, Ronowska A, Peksa R *et al*. Differentiation of high-risk stage I and II colon tumors based on evaluation of CAV1 gene expression. *J Surg Oncol* 2015; 112: 408-414.

36 Engelman JA, Zhang XL, Lisanti MP. Genes encoding human caveolin-1 and -2 are co-localized to the D7S522 locus (7q31.1), a known fragile site (FRA7G) that is frequently deleted in human cancers. *FEBS Lett* 1998; 436: 403-410.

37 Caruso M, Moore J, Goodall GJ, Thomas M, Phillis S, Tyskin A *et al*. Over-expression of cathepsin E and trefoil factor 1 in sessile serrated adenomas of

the colorectum identified by gene expression analysis. *Virchows Arch* 2009; 454: 291-302.

38 Delker DA, McGettigan BM, Kanth P, Pop S, Neklason DW, Bronner MP *et al*. RNA sequencing of sessile serrated colon polyps identifies differentially expressed genes and immunohistochemical markers. *PLoS One* 2014; 9: e88367.

39 Dunne PD, Dasgupta S, Blayney JK, McArt DG, Redmond KL, Weir JA *et al*. EphA2 Expression Is a Key Driver of Migration and Invasion and a Poor Prognostic Marker in Colorectal Cancer. *Clin Cancer Res* 2016; 22: 230-242.

40 Saito T, Masuda N, Miyazaki T, Kanoh K, Suzuki H, Shimura T *et al*. Expression of EphA2 and E-cadherin in colorectal cancer: correlation with cancer metastasis. *Oncol Rep* 2004; 11: 605-611.

41 Liu QZ, Gao XH, Chang WJ, Gong HF, Fu CG, Zhang W *et al*. Expression of ITGB1 predicts prognosis in colorectal cancer: a large prospective study based on tissue microarray. *Int J Clin Exp Pathol* 2015; 8: 12802-12810.

42 Hanski C, Hofmeier M, Schmitt-Gräff A, Riede E, Hanski ML, Borchard F *et al*. Overexpression or ectopic expression of MUC2 is the common property of mucinous carcinomas of the colon, pancreas, breast, and ovary. *J Pathol* 1997; 182: 385-391.

43 Tanaka H, Deng G, Matsuzaki K, Kakar S, Kim GE, Miura S *et al*. BRAF mutation, CpG island methylator phenotype and microsatellite instability occur more frequently and concordantly in mucinous than non-mucinous colorectal cancer. *Int J Cancer* 2006; 118: 2765-2771.

44 You JF, Hsieh LL, Changchien CR, Chen JS, Chen JR, Chiang JM *et al*. Inverse effects of mucin on survival of matched hereditary nonpolyposis colorectal cancer and sporadic colorectal cancer patients. *Clin Cancer Res* 2006; 12: 4244-4250.

45 Rademaker G, Costanza B, Bellier J, Herfs M, Peiffer R, Agirman F *et al*. Human colon cancer cells highly express myoferlin to maintain a fit mitochondrial network and escape p53-driven apoptosis. *Oncogenesis* 2019; 8: 21.

46 Park YY, Kim K, Kim SB, Hennessy BT, Kim SM, Park ES *et al*. Reconstruction of nuclear receptor network reveals that NR2E3 is a novel upstream regulator of ESR1 in breast cancer. *EMBO Mol Med* 2012; 4: 52-67.

47 Khanal T, Choi K, Leung YK, Wang J, Kim D, Janakiram V *et al*. Loss of NR2E3 represses AHR by LSD1 reprogramming, is associated with poor prognosis in liver cancer. *Sci Rep* 2017; 7: 10662.

48 Besson D, Pavageau AH, Valo I, Bourreau A, Bélanger A, Eymerit-Morin C *et al*. A quantitative proteomic approach of the different stages of colorectal

cancer establishes OLFM4 as a new nonmetastatic tumor marker. *Mol Cell Proteomics* 2011; 10: M111.009712.

49 Liu W, Liu Y, Zhu J, Wright E, Ding I, Rodgers GP. Reduced hGC-1 protein expression is associated with malignant progression of colon carcinoma. *Clin Cancer Res* 2008; 14: 1041-1049.

Reischmann_Supplementary Figure 1

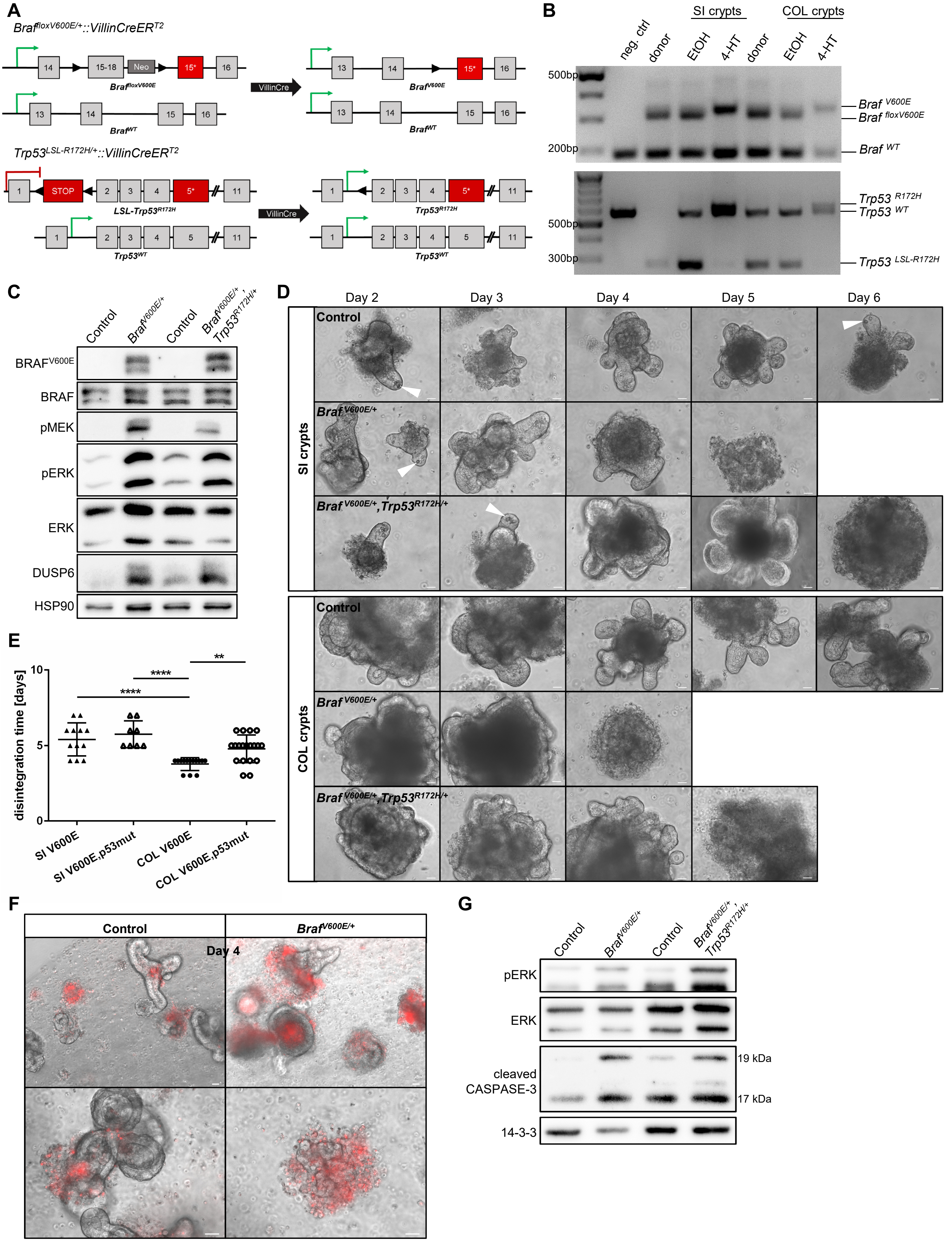

Reischmann_Supplementary Figure 2

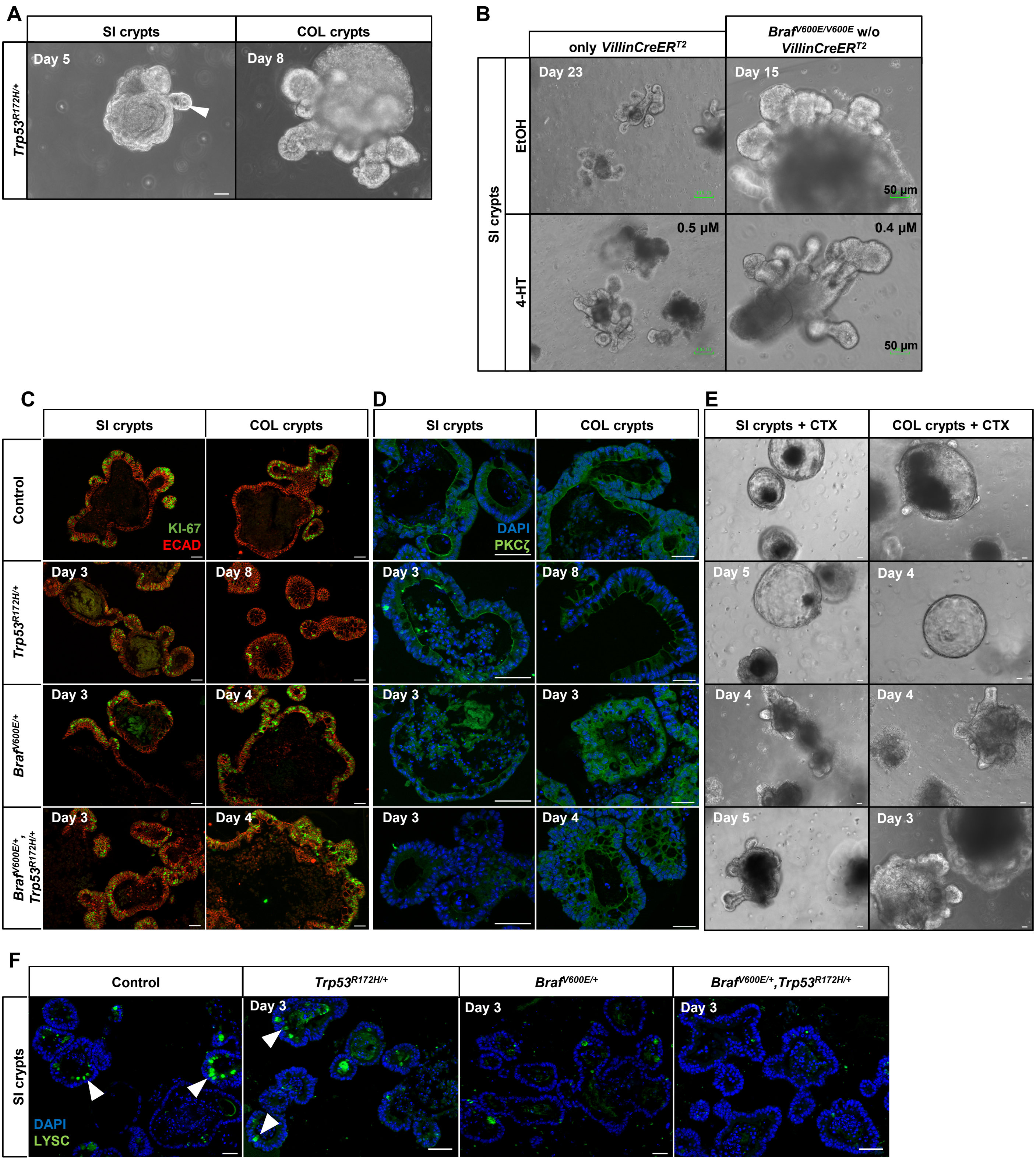

Reischmann_Supplementary Figure 3

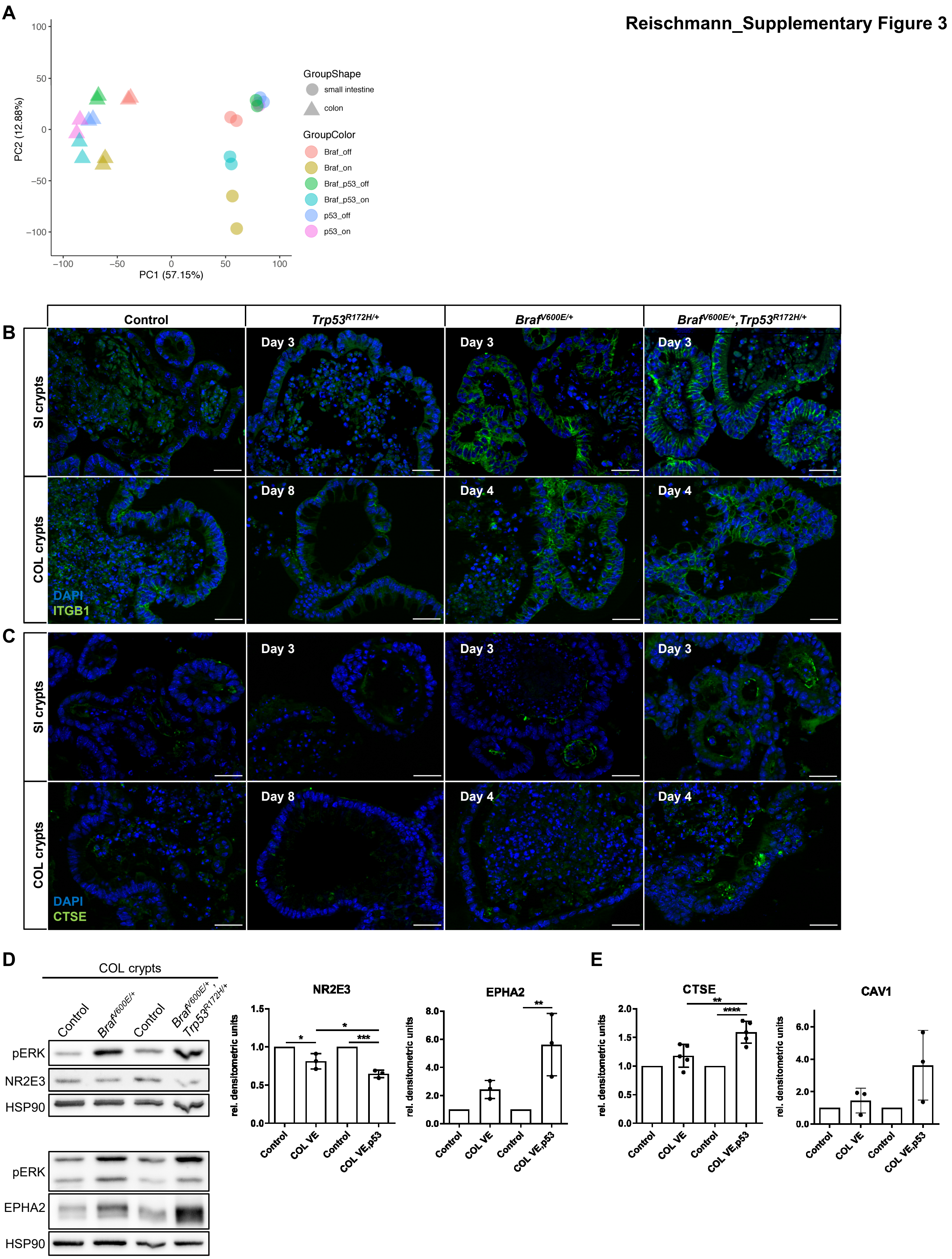

Reischmann_Supplementary Figure 4

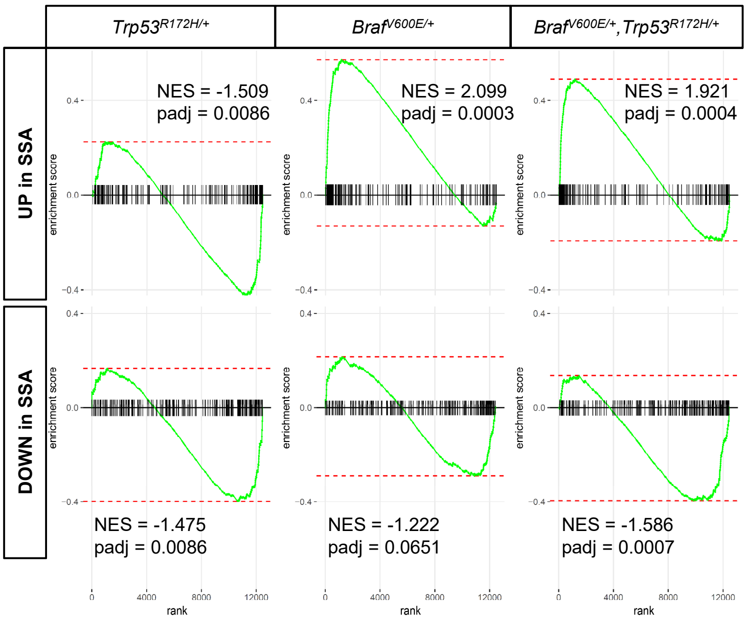

Reischmann_Supplementary Figure 5

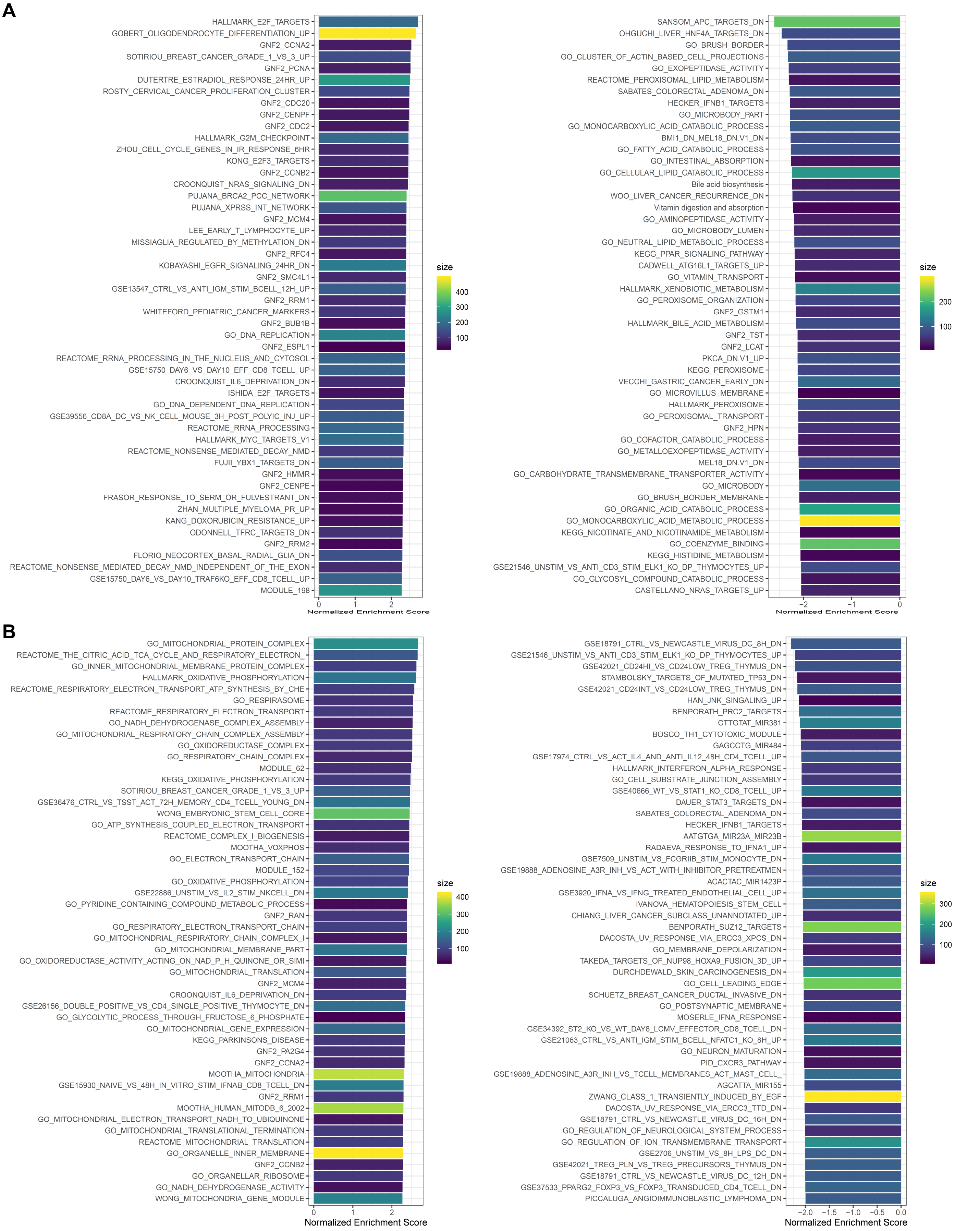

Reischmann_Supplementary Figure 6

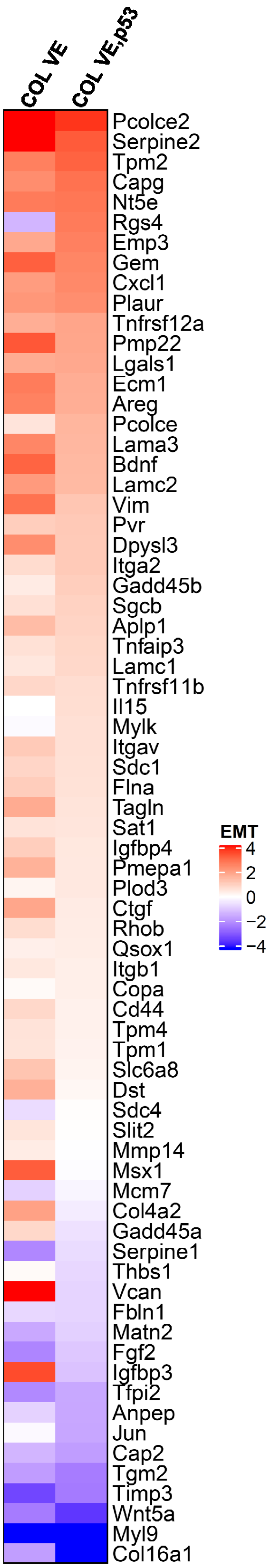

Reischmann_Supplementary Figure 7

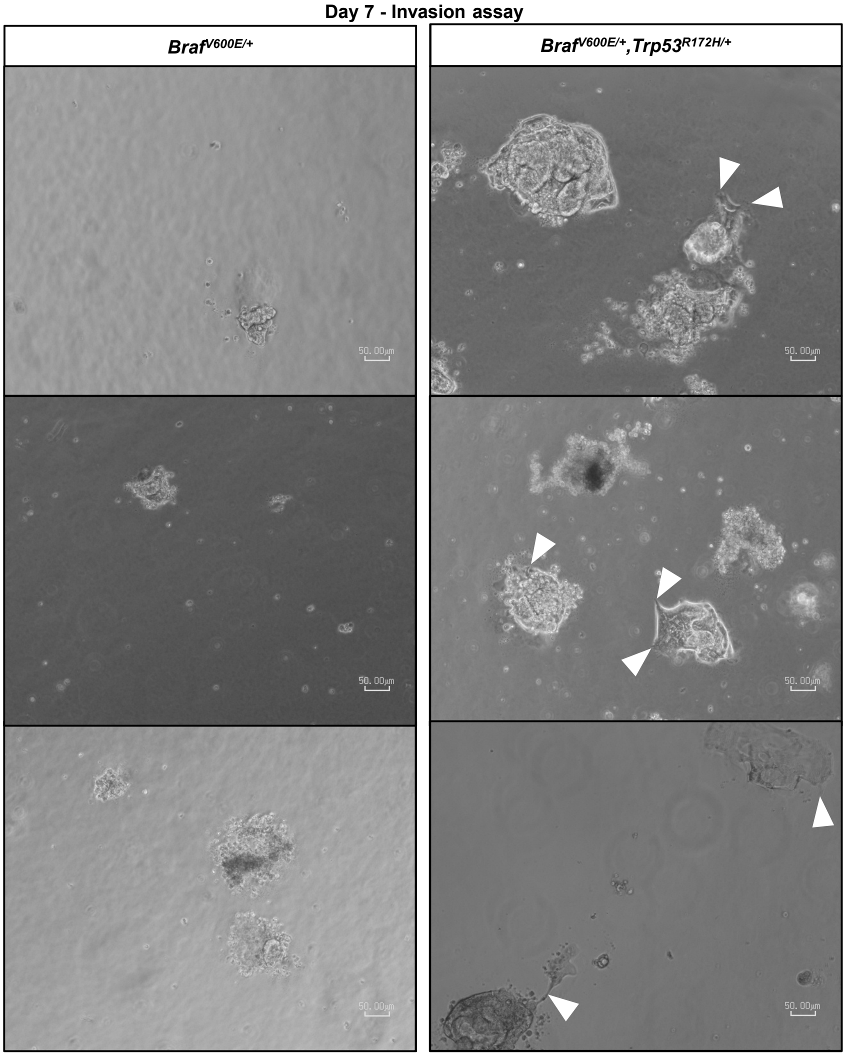

Reischmann_Supplementary Figure 8

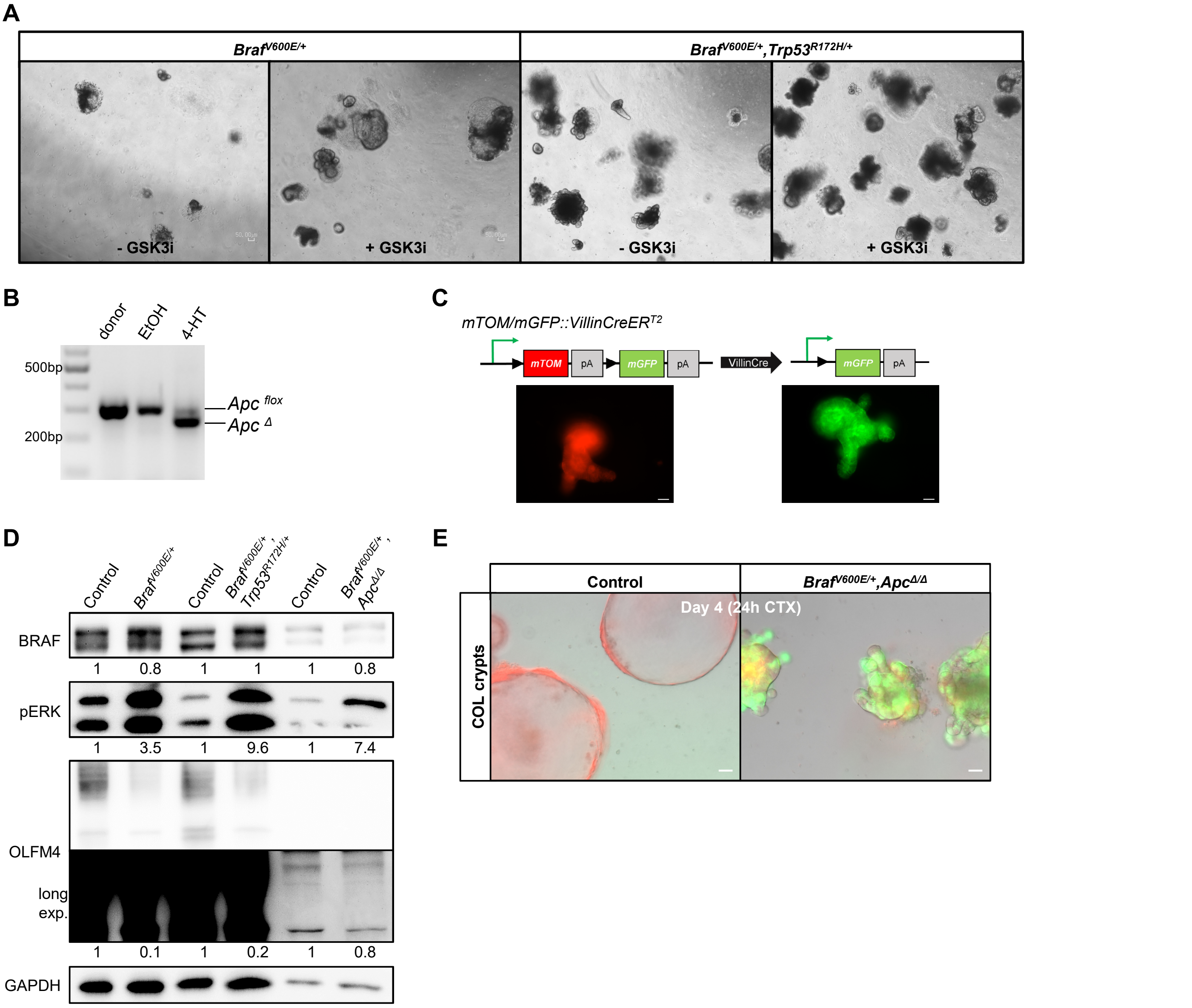

Reischmann_Supplementary Figure 9

A

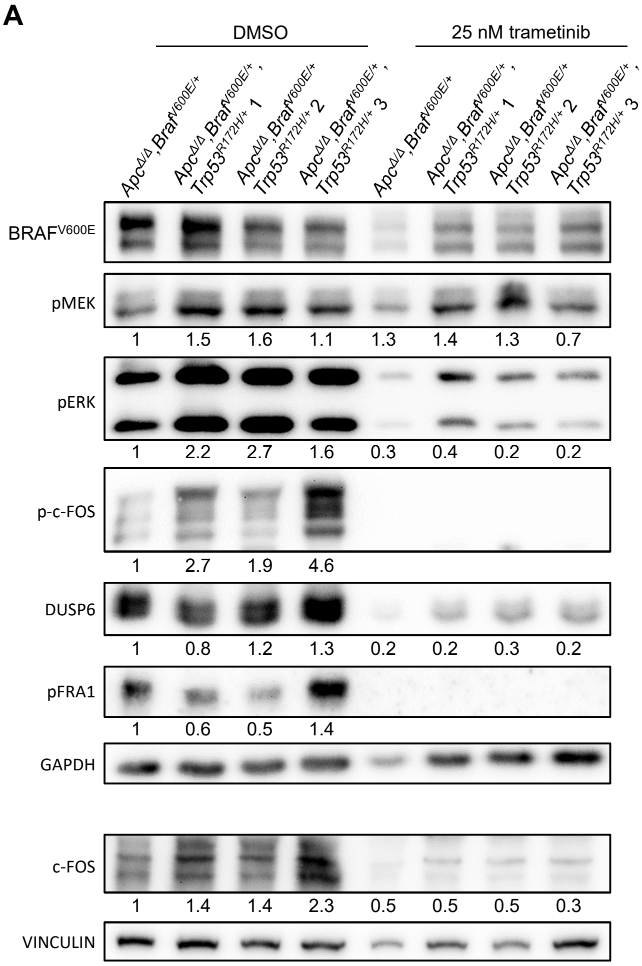

B

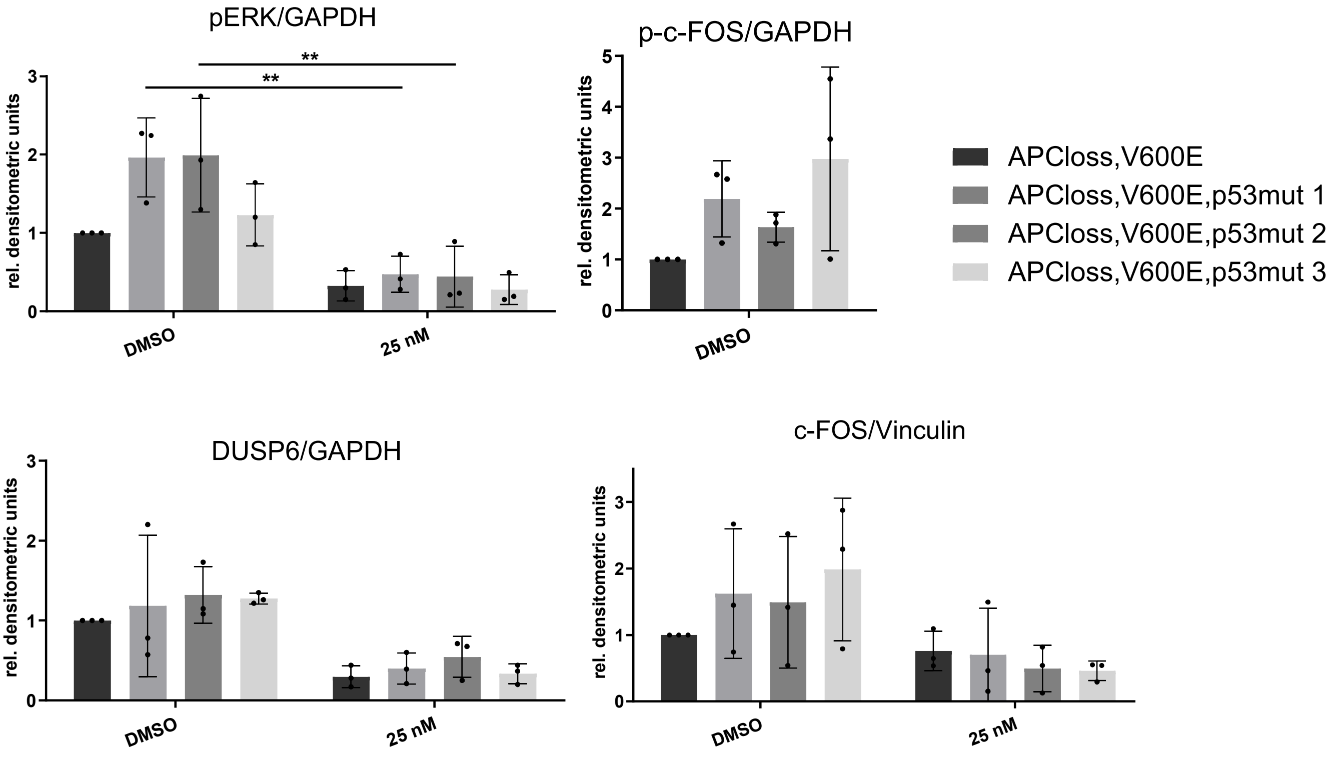

Reischmann_Supplementary Figure 10

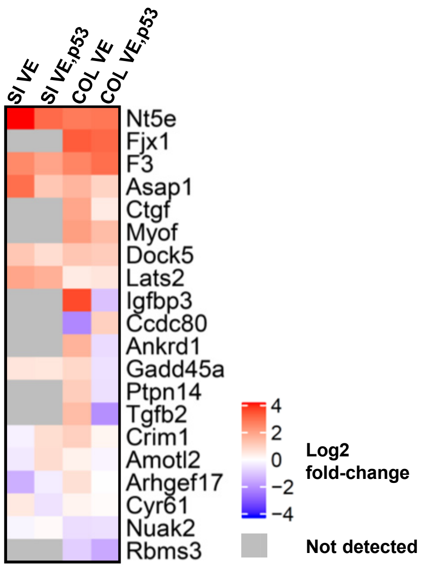

Supplement: Supplementary file 1 — Supplementary Materials and data [file 41388_2020_1414_MOESM1_ESM.pdf]
